# Adult antiretroviral therapy guidelines 2017

**DOI:** 10.4102/sajhivmed.v18i1.776

**Published:** 2017-07-15

**Authors:** Graeme Meintjes, Michelle A. Moorhouse, Sergio Carmona, Natasha Davies, Sipho Dlamini, Cloete van Vuuren, Thandekile Manzini, Moeketsi Mathe, Yunus Moosa, Jennifer Nash, Jeremy Nel, Yoliswa Pakade, Joana Woods, Gert Van Zyl, Francesca Conradie, Francois Venter

**Affiliations:** 1 Southern African HIV Clinicians Society, Johannesburg, South Africa

## Abstract

These guidelines are intended as an update to those published in the Southern African Journal of HIV Medicine in 2014 and the update on when to initiate antiretroviral therapy in 2015. Since the release of the previous guidelines, the scale-up of antiretroviral therapy (ART) in southern Africa has continued. New antiretroviral drugs have become available with improved efficacy, safety and robustness. The guidelines are intended for countries in the southern African region, which vary between lower and middle income.

## Key principles

While many antiretroviral therapy (ART) guidelines are available internationally, the current guidelines have been written to address issues relevant to southern Africa. The following general principles underpinned the writing process:

- Countries in the region vary between middle income and low-income countries; therefore, affordability was taken into account.
- Only treatment and diagnostic options available in southern Africa were included.
- We recognised the need to bridge the gap in treatment recommendations between public and private sector programmes, considering that many patients transition between the two sectors for treatment.
- While it is acknowledged that certain recommendations are aspirational for poorly resourced settings, the unavailability of diagnostic or monitoring tests should not pose a barrier to providing ART to those in need.

## Goals of antiretroviral therapy

The goals of ART are to:

- provide maximal and durable suppression of viral load (VL)
- restore and preserve immune function
- reduce HIV-related infectious and non-infectious morbidity
- prolong life expectancy and improve quality of life
- prevent onward transmission of HIV
- minimise adverse effects of the treatment.

These goals are achieved by suppressing viral replication completely for as long as possible, by using well tolerated and sustainable treatment taken with good adherence. With prolonged viral suppression, the CD4+ lymphocyte count usually increases, which is accompanied by a restoration of pathogen-specific immune function. For most patients, this results in a dramatic reduction in the risk of HIV-associated morbidity and mortality. For patients who start ART with preserved CD4+ counts, ART is able to prevent the decline in CD4+ count which has been observed in untreated patients and prevent clinical complications of HIV infection. It is still unclear whether immune function ever returns to full normality. Long-term cohorts show that patients who adhere well to ART have a near-normal life expectancy.^1^

## Standard of care

Maximally suppressive ART regimens should be used in the treatment of HIV-infected individuals to obtain the best results and to prevent resistance.

## Antiretroviral drugs: Classes and mechanisms of action

There are currently five classes of ART drugs available in southern Africa (Table 1). The most commonly used drugs inhibit one of three key HIV enzymes required by the virus for intracellular replication:

- Reverse transcriptase: essential for completion of the early stages of HIV replication.
- Integrase: required for the integration of proviral DNA into the host chromosomal DNA.
- Protease: required for the assembly and maturation of infectious viral progeny.

**TABLE 1:** Classes of antiretroviral agents.

Class	Abbreviation	Mechanism of action	Specific action
Nucleoside and nucleotide reverse transcriptase inhibitors	NRTIs and NtRTIs	Reverse transcriptase inhibition	Nucleic acid analogues mimic the normal building blocks of DNA, preventing transcription of viral RNA to DNA
Non-nucleoside reverse transcriptase inhibitors	NNRTIs	Reverse transcriptase inhibition	Alter the conformation of the catalytic site of reverse transcriptase and directly inhibit its action
Protease inhibitors	PIs	Protease inhibition	Inhibit the final maturation stages of HIV replication, resulting in the formation of non-infective viral particles
Integrase inhibitors (also termed integrase strand transfer inhibitors)	InSTIs	Inhibition of viral integration	Prevent the transfer of proviral DNA strands into the host chromosomal DNA
Entry inhibitors	–	Entry inhibition	Bind to viral gp41 or gp120 or host cell CD4+ or chemokine (CCR5) receptors

CCR5, C-C chemokine receptor type 5; NRTIs, nucleoside reverse transcriptase inhibitors; NtRTIs, nucleotide reverse transcriptase inhibitors; NNRTIs, non-nucleoside reverse transcriptase inhibitors; PIs, protease inhibitors; InSTIs, integrase inhibitors (integrase strand transfer inhibitors).

## Antiretroviral drugs currently available in southern Africa

The antiretroviral (ARV) drugs currently available in southern Africa are summarised in Table 2. A number of two-drug and three-drug fixed dose combinations (FDCs) are available in the region. These FDCs reduce the burden of multiple pills and may improve treatment adherence.

**TABLE 2:** Dosage and common adverse drug reactions of antiretroviral drugs available in southern Africa.

Generic name	Class of drug‡	Recommended dosage	Common or severe ADR§
Tenofovir (TDF)¶	NtRTI	300 mg daily	**Renal failure**, tubular wasting syndrome, reduced bone mineral density, nausea
Lamivudine (3TC)	NRTI	150 mg 12-hourly or 300 mg daily	**Anaemia (pure red cell aplasia)** (rare)
Emtricitabine (FTC)†	NRTI	200 mg daily	Palmar hyperpigmentation
Abacavir (ABC)	NRTI	300 mg 12-hourly or 600 mg daily	**Hypersensitivity reaction**
Zidovudine (AZT)¶	NRTI	300 mg 12-hourly	**Anaemia, neutropenia,** GI upset, headache, myopathy, hyperlactataemia or steatohepatitis (medium potential), lipoatrophy
Stavudine (d4T)¶	NRTI	30 mg 12-hourly	Peripheral neuropathy, lipoatrophy, **hyperlactataemia** or steatohepatitis (high potential), **pancreatitis**, dyslipidaemia
Didanosine (ddI)¶	NRTI	400 mg daily (250 mg daily if < 60 kg) taken on an empty stomach (only enteric-coated formulation available)	Peripheral neuropathy, **pancreatitis,** nausea, diarrhoea, **hyperlactataemia** or steatohepatitis (high potential)
Efavirenz (EFV)	NNRTI	600 mg at night (400 mg at night if < 40 kg)	Central nervous system symptoms (vivid dreams, problems with concentration, dizziness, confusion, mood disturbance, psychosis), **rash, hepatitis,** gynaecomastia
Nevirapine (NVP)	NNRTI	200 mg daily for 14 days, then 200 mg 12-hourly	**Rash,** hepatitis
Rilpivirine (RPV)	NNRTI	25 mg daily with food	**Rash, hepatitis,** central nervous system symptoms (all uncommon)
Etravirine (ETR)	NNRTI	200 mg 12-hourly	Rash, hepatitis (both uncommon)
Atazanavir (ATV)	PI	400 mg daily (only if ART-naive) or 300 mg with ritonavir 100 mg daily (preferable) with TDF, always 300/100 mg daily and with EFV 400/100 mg daily	Unconjugated hyperbilirubinaemia (visible jaundice in minority of patients), dyslipidaemia (low potential), renal stones (rare), hepatitis (uncommon)
Lopinavir/ritonavir (LPV/r)	Boosted PI	400/100 mg 12-hourly or 800/200 mg daily (only if PI-naive)	GI upset, dyslipidaemia, hepatitis
Darunavir (DRV)	PI	600 mg 12-hourly with 100 mg ritonavir 12-hourly or 800/100 mg daily (only if PI-naive)	GI upset, rash, dyslipidaemia, hepatitis (uncommon). Contains sulphonamide moiety (use with caution in patients with sulpha allergy)
Saquinavir (SQV) (rarely used)††	PI	1000 mg with 100 mg ritonavir; 12-hourly, or 1600 mg with 100 mg ritonavir daily (only if PI-naive); Take with a fatty meal, or up to 2 h after meal	GI disturbance (mild), hepatitis, hyperglycaemia, dyslipidaemia
Raltegravir (RAL)	InSTI	400 mg 12-hourly	Headache and other CNS side effects, GI upset, hepatitis and rash (rare), rhabdomyolysis (rare)
Dolutegravir (DTG)	InSTI	50 mg daily	Insomnia, headache and other CNS side effects, GI upset, hepatitis and rash (rare)
Maraviroc (MVC)	CCR5 blocker	150 mg, 300 mg or 600 mg 12-hourly (doses depend on concomitant medication and interactions)	Rash, hepatitis, fever, abdominal pain, cough, dizziness, musculoskeletal symptoms (all rare)

Note: Patients receiving SQV should be switched to the recommended PIs (consult an expert if the patient’s VL is not suppressed). We recommend against regimens containing dual RTV-boosted PIs, as there is no evidence for superior efficacy^2^ and more side effects are likely. Low-dose RTV is used to ‘boost’ the concentration of other PIs. It is always used with LPV (in FDC) and is strongly encouraged with all other PIs. In rare situations, ATV without boosting is used in first-line therapy.

Efavirenz 400 mg nocte is non-inferior in terms of virological efficacy to 600 mg nocte regardless of weight, but this dose has not been evaluated in patients who are pregnant and patients on tuberculosis treatment.

We now discourage the use of d4T, ddI or NVP unless there is a specific clinical reason (e.g. intolerance of multiple other drugs) given the toxicity associated with these drugs and the availability of alternatives. D4T, ddI and SQV are now rarely used.

ARV, antiretroviral; ADR, adverse drug reaction; NtRTI, nucleotide reverse transcriptase inhibitor; NRTI, nucleoside reverse transcriptase inhibitor; SA, South Africa; GI, gastrointestinal; NNRTI, non-nucleoside reverse transcriptase inhibitor; PI, protease inhibitor; ART, antiretroviral therapy; InSTI, integrase inhibitor (integrase strand transfer inhibitor); CCR5, C-C chemokine receptor type 5; RTV, ritonavir; FDC, fixed dose combination; VL, viral load; TDF, tenofovir; DRV/r, darunavir/ritonavir.

† , FTC is not available as a single drug in SA, only co-formulated; ‡, All PIs may be associated with cardiac conduction abnormalities (especially PR interval prolongation). This seldom results in clinically significant effects, but caution should be taken when co-prescribing other drugs that cause delayed cardiac conduction, such as macrolides; §Life-threatening reactions are indicated in bold; ¶, ARV combinations to be avoided include:
AZT + d4T (antagonism) TDF + ddI (associated with poorer virological and immunological responses and increased toxicity) d4T + ddI (associated with a very high risk for mitochondrial toxicities such as lactic acidosis and peripheral neuropathy) ETR + ATV/r (due to drug interaction) ETR + DTG unless a boosted PI is also used in the combination (due to drug interaction)

†† , The following three PIs are recommended for use: LPV/r, ATV/r and DRV/r.

## Indications for initiating antiretroviral therapy

We now advise starting ART in all individuals diagnosed with HIV. In general, delays to starting ART should be minimised. In particular, patients with profound immunosuppression (CD4+ count < 200 cells/μL) are at significant risk of opportunistic infections (OIs) and associated mortality, and should be assessed rapidly and initiated on ART within one week once adherence counselling has been initiated. An exception to this is patients presenting with cryptococcal meningitis (CM) or tuberculous meningitis (TBM) – see below.

### Rationale for these guidelines

In 2015, two seminal randomised controlled trials (RCTs) that addressed the optimal timing of ART in HIV-infected patients with high CD4+ counts were published: strategic timing of antiretroviral therapy (START) and TEMPRANO ANRS 12136 (early antiretroviral treatment and early isoniazid prophylaxis against tuberculosis in HIV-infected adults).^3,4^ The findings of these two trials were consistent: there was significant individual clinical benefit from starting ART immediately in patients with CD4+ counts higher than 500 cells/μL rather than deferring until a certain lower CD4+ threshold or clinical indication was met.

### Benefits of antiretroviral therapy in reducing transmission

There is evidence that starting ART when HIV-positive people have higher CD4+ counts reduces HIV transmission within couples where one partner is HIV-negative (HPTN 052). This trial showed that treating the HIV-positive partner in a serodiscordant relationship with ART was associated with a 93% reduction in transmission risk to the uninfected partner.^5,6^ Wider ART coverage appears to reduce the risk of HIV transmission at a community level. Therefore, early ART initiation has the public health benefits of potentially reducing both HIV incidence and morbidity. This led the Southern African HIV Clinicians Society to update its guidelines on starting ART in 2015 to advise starting ART in all patients diagnosed with HIV infection regardless of CD4+ count or symptoms.

### Commencing antiretroviral therapy in patients with tuberculosis

Decisions regarding the timing of ART in patients with TB should be made on the basis of the CD4+ count.

#### CD4+ count ≤ 50 cells/µL

Antiretroviral therapy should be regarded as urgent, with the aim to start therapy two weeks following the commencement of TB treatment. A meta-analysis of RCTs has demonstrated that this approach reduces mortality.^7^ It is advised to commence ART after it is clear that the patient’s TB symptoms are improving and that TB therapy is tolerated. The exception to this is in the case of CM (see the section ‘Starting antiretroviral therapy in patients with other opportunistic infections and acute illnesses’) or TBM.

#### CD4+ count > 50 cells/µL

Antiretroviral therapy can be delayed until eight weeks after starting TB treatment, but no later. However, if the patient has other WHO stage 4 conditions, ART should also be initiated two weeks after TB treatment is started. The exception to this is in the case of CM (see the section ‘Starting antiretroviral therapy in patients with other opportunistic infections and acute illnesses’) or TBM. The longer delay before commencing ART in this group is anticipated to reduce the risk of shared toxicity (as the patient will then be receiving fewer TB drugs) and to reduce the risk of immune reconstitution inflammatory syndrome (IRIS) (see the section ‘Immune reconstitution inflammatory syndrome’). The aforementioned meta-analysis of RCTs did not show a higher risk of AIDS progression or mortality in this group when ART initiation was delayed until approximately eight weeks after starting TB treatment.^7^

#### Tuberculous meningitis

In patients with TBM, starting ART immediately or at two months following diagnosis was shown to have similar high mortality, with more complications in the immediate group.^8^ We recommend starting ART 4–8 weeks after TBM diagnosis.

There are important drug interactions and shared side effects when ART is co-administered with TB therapy (see the section ‘Tuberculosis’). When ART is commenced, patients should be warned that TB symptoms or signs may temporarily worsen and new features may occur in the first three months as a result of TB-IRIS (see the section ‘Immune reconstitution inflammatory syndrome’).

### Starting antiretroviral therapy in patients with other opportunistic infections and acute illnesses

With most OIs and acute illnesses (e.g. pneumocystis or bacterial pneumonia), the clinician should aim to start ART within two weeks of commencing treatment for that infection.^9^ In patients with severe Kaposi’s sarcoma and lymphoma, ART counselling should be expedited and ART should be initiated as soon as possible. In a patient diagnosed with an OI in hospital, it is important to ensure referral and linkage to outpatient services for ART initiation without delay. For patients with CM, the optimal time to start ART is 4–6 weeks from the time of starting CM treatment. The COAT (Cryptococcal Optimal ART Timing) trial demonstrated a significantly higher mortality in patients who started ART in hospital 1–2 weeks after CM diagnosis than in those starting 5–6 weeks after diagnosis.^2^

In HIV-infected patients admitted to the intensive care unit (ICU), if the patient is receiving ART, then this should be continued (through nasogastric tube [NGT], if necessary) and only interrupted if the gastrointestinal tract is not functional (e.g. ileus). If the patient is not yet receiving ART, then it should not be commenced if the reason for admission is an acute critical illness or injury. There are several potential problems associated with commencing ART in this setting: lack of adequate counselling, gastrointestinal dysfunction, malabsorption and possible development of resistance. There are no intravenous options for an ART regimen. In patients admitted to the ICU for prolonged periods, ART initiation in the unit should be considered after multi-organ failure has resolved. Certain ART preparations should not be administered via NGT. In general, paediatric syrups can be administered via NGT. A pharmacist should always be consulted regarding which ART drugs can be administered via NGT and how to do this.

### Patient readiness for antiretroviral therapy

Preparing patients for lifelong ART with good adherence is a critical component of achieving long-term efficacy and preventing resistance:

- To accommodate counselling, traditionally two or three visits were required, staggered closely together, either before or during early ART (see the section ‘Commencing antiretroviral therapy at the first antiretroviral therapy clinic visit’, where same-day initiation may be an option). Prolonged delays in commencing ART should be avoided. ART should be delayed only if concerns about adherence are severe enough to outweigh the risk of HIV disease progression.
- The patient should be provided with details on the following:
  ■ the benefits of ART
  ■ that ART is a lifelong therapy
  ■ the importance of good adherence
  ■ list of ART side effects relevant to the drugs they will use, including what to do and who to contact if serious side effects occur.
- Counselling should also ensure that the patient has a good understanding of HIV (the virus, the potential clinical complications and transmission) and should cover safer-sex practices and address issues related to reproductive health (i.e. family planning, contraception, condom use and pregnancy). Clinicians should check family planning choices at follow-up visits too and adequate access to safe and effective contraception should be provided.
- Active depression, other mental health issues or substance abuse should actively be detected and treated.
- A personal treatment plan should be formulated for each patient, specifying drug storage, strategies for missed doses and how to integrate taking medication into their daily routine. The patient must be made aware of scheduling in terms of clinical follow-up.
- Disclosure of HIV status (to a partner or other household members) should strongly be encouraged. This has been shown to be an important determinant of treatment adherence and assists in the provision of patient-directed support. Disclosure also identifies exposed contacts for screening and support. This issue needs to be handled sensitively in situations where disclosure may have harmful consequences, particularly for women.
- The patient should be encouraged to join a support group or identify a treatment ‘buddy’. However, neither disclosure nor support group participation is a prerequisite for good adherence in all patients, and should not be a reason for deferring ART.
- Clinicians should ensure that they have the contact details of each patient and their treatment buddy.

### Commencing antiretroviral therapy at the first antiretroviral clinic visit

Several studies have demonstrated that it is possible to initiate ART safely on the same day as HIV diagnosis or receipt of CD4+ count result.^10,11,12^ These studies have demonstrated less overall loss to follow-up when ART is initiated immediately in selected patients. Now that treatment is recommended irrespective of CD4+ count this same-day strategy should be considered as a means to improve retention in care.

More experience is needed to make firm recommendations, and this approach is being tested in different programmes. Until then, the following should be considered for patients newly diagnosed or returning for CD4+ or other test results, when deciding about initiating ART on the same day:

- The patient should be motivated to start immediately.
- Same-day initiation is not an adherence support ‘short cut’; ongoing support can occur in the days and weeks immediately after initiation.
- Patients starting tenofovir (TDF) (which are the majority) should be contactable in the event of a creatinine clearance (CrCl) < 50 mL/min, and told to return to the clinic immediately.
- A serum or plasma CrAg should be conducted in those with CD4+ < 100 cells/μL; again, the patient should be contactable in the event of a positive CrAg and must be advised to return to the clinic immediately.
- Symptom screening for TB and CM before initiation of treatment is important. Patients with TB symptoms (e.g. cough, night sweats, fever and recent weight loss) should first be investigated for TB before ART initiation. Likewise in a patient with a headache, meningitis should be considered and investigated if appropriate (i.e. lumbar puncture [LP]) before starting ART. Table 3 lists the medical reasons for deferring ART initiation.

**TABLE 3:** Medical reasons to defer initiation of antiretroviral therapy.

Reason	Action
Diagnosis of CM	Defer ART for 4–6 weeks after start of antifungal treatment
Serum or plasma cryptococcal antigen positive	Defer ART for 2 weeks after start of antifungal treatment (if meningitis is excluded on LP then ART does not need to be deferred)
Diagnosis of TB meningitis or tuberculoma	Defer ART until 4–8 weeks after start of TB treatment
Diagnosis of TB at non-neurological site	Defer ART up to 2 weeks after start of TB treatment if CD4^+^ ≤ 50 cells/μL and up to 8 weeks if CD4^+^ > 50 cells/μL
Headache	Investigate for meningitis before starting ART
TB symptoms (cough, night sweats, fever, recent weight loss)	Investigate for TB before starting ART
Significantly abnormal liver function tests (ALT > 200 or jaundice)	Investigate and address the cause before starting ART, including other drugs causing DILI

CM, cryptococcal meningitis; ART, antiretroviral therapy; TB, tuberculosis; ALT, alanine transaminase; DILI, drug-induced liver injury; LP, lumbar puncture.

### Antiretroviral therapy in primary HIV infection

For patients who are diagnosed with HIV during acute seroconversion, we advise that those patients be counselled and initiated on ART as soon as possible. This should preferably be expedited ART initiation as there is evidence that this may limit the size of the HIV reservoir.^13^ Previously, we suggested that these patients could have ART interrupted. Rather, once ART is started in this situation, this should be lifelong ART, and this should be discussed with the patient. Additional counselling once the patient is established on ART may be required for patients who start ART in this acute situation because there is limited time for extensive counselling pre-ART, and there is often considerable psychological distress around this time. Diagnosing seroconversion is facilitated by having a recent negative HIV test that then becomes positive on a subsequent test. Otherwise, the following are suggestive: the compatible clinical syndrome, an indeterminate enzyme-linked immunosorbent assay (ELISA) test result that then becomes positive on a subsequent test, and a very high VL.

### Antiretroviral therapy initiation in ‘elite controllers’

A minority of patients (< 1%) have very effective immune control of HIV infection and are able to control HIV viraemia at undetectable levels in the absence of ART – termed ‘elite controllers’. An argument could be made that such individuals do not require ART if their CD4+ count is > 500 cells/μL. The START and TEMPRANO trials did not specifically address the question of whether early ART was beneficial in such patients, and it is highly unlikely that any sufficiently powered RCT could ever be conducted focused on these individuals. In the absence of such data, we rely on indirect evidence. Firstly, elite controllers still have evidence of chronic immune activation and inflammation that may drive non-infectious morbidities.^14^

Secondly, elite controllers have been shown to have a higher rate of hospitalisation than patients who are virologically controlled by ART.^15^ For these reasons, we do advise starting ART in elite controllers too, with the same caveats regarding the patient being prepared.

One important consideration in such patients is that careful attention should be paid to confirming the diagnosis of HIV before starting ART. They are typically patients who have a positive HIV ELISA test, undetectable HIV VL, CD4+ count in the normal range and are clinically well. The possibility of a false-positive HIV ELISA test should be excluded either by performing a qualitative HIV DNA polymerase chain reaction assay or a Western blot assay. If the patient at some point previously had a detectable HIV VL, this would also serve as confirmation. Such patients should be discussed with a laboratory virologist to assist with confirmation of HIV infection status.

## Investigations prior to starting antiretroviral therapy

If HIV diagnosis has been made by using two rapid tests performed outside of a laboratory setting, then we advise confirming the positive serostatus by using a laboratory test prior to commencing lifelong ART. A detectable VL result would be sufficient (note that it may be undetectable in < 1% of patients not receiving ART, i.e. ‘elite controllers’), but if unaffordable or unavailable, then an ELISA should be performed.

The following investigations are recommended prior to initiating ART:

- alanine transaminase (ALT)
- full blood count (FBC) if AZT being considered: avoid AZT if haemoglobin (Hb) is < 8 g/dL
- serum creatinine and calculation of CrCl: avoid TDF if CrCl is < 50 mL/min; other nucleoside reverse transcriptase inhibitors (NRTIs), except abacavir (ABC), require dose adjustment if CrCl is < 50 mL/min (either by using the estimated glomerular filtration rate [eGFR] provided by the laboratory or calculating by using the modified Cockgraft–Gault equation, Table 11)
- hepatitis B surface antigen (HBsAg – see the section ‘Hepatitis B co-infection’)
- CD4+ count
- baseline VL
- syphilis serology
- serum cryptococcal antigen test in patients starting ART at a CD4+ count < 100 cells/μL (to screen for early cryptococcal disease and to initiate pre-emptive treatment if positive)

We advise a symptom screen for TB (cough, weight loss, fever, night sweats). If any are present then send sputum for Xpert and TB culture, and if CD4+ < 100 cells/μL then send urine for lipoarabinomannan (LAM) assay.

## Initial antiretroviral therapy regimens for the previously untreated patient

The preferred first-line regimens are (see Table 4):

- TDF + emtricitabine (FTC) (or 3TC) + efavirenz (EFV) or
- TDF + emtricitabine (FTC) (or 3TC) + dolutegravir (DTG) or
- TDF + emtricitabine (FTC) (or 3TC) + rilpivirine (RPV) provided VL < 100 000 copies/mL.

**TABLE 4:** Preferred first-line regimen options.

Options	Preferred	Alternative	One of
NRTI backbone	TDF + FTC/3TC	ABC† + 3TC	–
	–	AZT‡ + 3TC	–
	–	d4T§ + 3TC	–
Third drug	–	–	EFV
	–	–	DTG
	–	–	RPV¶

NRTI, nucleoside reverse transcriptase inhibitor; tenofovir; FTC, emtricitabine; 3TC, lamivudine; ABC, abacavir; AZT, zidovudine; d4T, stavudine; EFV, efavirenz; DTG, dolutegravir; RPV, rilpivirine.

† , If creatinine clearance < 50 mL/min; ‡, Only if both TDF and ABC contraindicated or unavailable AND haemoglobin > 8 g/dL; §, Only for short-term use in patients with contraindications to all other NRTIs – we advise against using d4T for longer than 3 months; ¶, Only if VL < 100 000 copies/mL.

Rilpivirine cannot be used with rifampicin, and dolutegravir requires dose adjustment with rifampicin.

### Nucleoside reverse transcriptase inhibitor component of first-line regimen

The recommended NRTI drugs are TDF with FTC (emtricitabine) or 3TC. FTC and 3TC are both co-formulated in two-drug FDCs with TDF, and in three-drug FDCs with TDF and EFV.

We favour regimens that include FDCs and allow once-daily dosing. TDF is the favoured NRTI to use with 3TC or FTC, as it aligns with public sector programmes, is widely available as an FDC and is very well tolerated. However, patients with a CrCl < 50 mL/min should not start TDF and should rather start ABC. A meta-analysis showed that virological suppression is equivalent with ABC- and TDF-containing first-line regimens regardless of baseline VL.^16^ ABC has been associated with an increased risk of myocardial infarction in some but not other cohort studies,^17,18^ but the association was not confirmed in a meta-analysis of RCTs.^19^ Nevertheless, caution is recommended when considering ABC for patients at significant risk of, or with established ischaemic heart disease. ABC, which does not require dose adjustment in renal failure, is specifically recommended for use during chronic renal failure, as TDF is nephrotoxic and AZT could aggravate the anaemia of renal failure. We now recommend that AZT or short-term stavudine (d4T) is only used in special circumstances in first-line therapy. If both TDF and ABC are unavailable or contraindicated, then AZT should be used, provided that the Hb level is > 8 g/dL. As it is considerably more toxic than other NRTIs, d4T is no longer recommended. Nonetheless, there is still a role for d4T in selected patients, when it is used in the short-term in patients with contraindications to other NRTIs. A common example is a patient with renal dysfunction and anaemia at baseline, who could be initiated on d4T for three months if ABC is unavailable, and then switched to AZT or TDF depending on resolution of the anaemia or renal dysfunction. Patients usually tolerate short-term d4T (≤ 3 months) well. Severe d4T side effects, such as hyperlactataemia, lipoatrophy and other mitochondrial toxicities, typically occur after six months, although peripheral neuropathy can develop earlier.

If there is a need for concomitant nephrotoxic medications, e.g. aminoglycosides to treat multidrug-resistant (MDR)-TB, then ABC or AZT is preferable to TDF during the period of exposure to the other nephrotoxic medication.

### The third drug in the regimen: Efavirenz, dolutegravir or rilpivirine

The WHO guidelines currently recommend efavirenz-based first-line ART, with EFV 600 mg as the preferred option and EFV 400 mg as an alternative option. EFV 600 mg is available in public sector programmes in most countries in southern Africa. Efavirenz is thus very widely used in first-line regimens in the region. Although EFV 400 mg demonstrated non-inferior efficacy with moderately improved tolerability in ENCORE1,^20^ this reduced dose has not been studied in pregnant women or patients who are receiving rifampicin-based TB treatment. EFV 400 mg is currently also not available in FDCs, and for these reasons, we do not recommend the routine use of EFV 400 mg in first-line ART. Dolutegravir is now available as an alternative for first-line ART in the private sector, is included in the first-line regimen in the public sector in Botswana and is likely to be accessible in public sectors in other countries in the region when affordable generic options become available at scale.

Dolutegravir has been shown to be superior to efavirenz-based ART in the SINGLE trial and also has a higher barrier to resistance. The SINGLE trial compared DTG/ABC/3TC versus EFV/TDF/FTC in ART-naïve adults in North America, Europe and Australia (*n* = 833). At week 48, the proportion of participants with a VL < 50 copies/mL was 88% in the DTG arm versus 81% in the EFV arm (*p* = 0.003). This difference was largely driven by the superior tolerability of the DTG arm: 2% vs 10% on the EFV arm had an adverse event leading to discontinuation of study drug.^21^ In the 144-week results of this trial the DTG arm remained superior: 71% vs 63% maintained VL suppression < 50 copies/mL (*p* = 0.01). A striking result was that no participants in the DTG/ABC/3TC arm developed treatment-emergent integrase or NRTI resistance mutations over three years, demonstrating the high barrier to resistance of a DTG-containing first-line regimen.^22^

Dolutegravir may cause a small increase in serum creatinine because of interference with tubular secretion. This does not represent renal damage and is not an indication for switching off the drug. There are currently limited data on the use of DTG in patients being treated for TB and during pregnancy, but trial data on these groups are expected soon.

Dolutegravir is preferred to the other integrase inhibitor available in southern Africa, raltegravir (RAL), the major reason being that it has a higher barrier to resistance. In the SPRING-2 trial, DTG- and RAL-containing first-line regimens were compared: DTG was found to be non-inferior.^23^ At week 48, 88% of patients on DTG achieved virological suppression (VL < 50 copies/mL) vs 85% on RAL (difference not significant). Adverse events were similar between treatment groups. No patients in the DTG arm were found to have developed resistance, whereas, in patients in the RAL arm, one developed integrase resistance and four developed NRTI resistance. The high barrier to resistance of DTG-containing ART regimens has been replicated in other first-line studies and in a study of ART-experienced patients in which DTG was compared to RAL.^22,24,25^ In a meta-analysis that included clinical trials and observational studies, the emergence of integrase resistance was more common with RAL than with DTG (3.9% vs 0.1%).^26^ In addition, the emergence of integrase resistance on RAL compromises second-generation InSTIs (which otherwise have a very high barrier to resistance) in a proportion of cases.

Another option in first-line regimens is RPV, a second-generation NNRTI. RPV is inexpensive, but drawbacks are that it cannot be used with rifampicin-based TB treatment (subtherapeutic concentrations because of rifampicin induction of its metabolism), and it should not be started in a patient with a VL > 100 000 copies/mL (it is inferior to EFV in such patients).^27^ It is also not yet available in an FDC in the region.

We no longer recommend NVP use for new patients starting ART because of the severe toxicity that may be associated with its use. In patients currently tolerating NVP, there is no reason to switch for toxicity, which characteristically occurs in the first three months of taking NVP and not later. However, switch for simplification to a once-daily regimen, providing that there is virological suppression, should be considered.

For patients transitioning from the private sector on DTG first-line to the public sector and requiring a switch because DTG is unavailable, we recommend doing a VL and switching to EFV. If VL < 50 copies/mL then repeat VL in one year. If VL is not suppressed then switch to EFV, counsel regarding adherence and repeat VL in 2–3 months.

### Baseline resistance test

We recommend a baseline resistance test to guide first-line regimen choice only in the following situations:

- pre-exposure prophylaxis (PrEP) received in the previous 6 months
- history of sexual exposure to a person with known drug-resistant HIV or known to have failed an ART regimen.

## Laboratory monitoring for antiretroviral therapy efficacy and safety

### Viral load

#### Timing of viral load monitoring

Viral load monitoring should be performed:

- at baseline (before commencing ART)
- at three months after the commencement of ART (This early VL is desirable to detect adherence problems early, before resistance develops. A subset of patients who start with a very high VL may not be fully suppressed at three months despite 100% adherence, but such patients would have had a > 2 log10 drop in VL from baseline if adherence is optimal and there is no resistance. Therefore, the 3-month result should be interpreted in relation to the baseline VL. All patients who have a detectable VL at three months should receive additional adherence interventions.)
- at six months and thereafter every six months (In patients who have an undetectable VL for > 12 months, and who demonstrate reliable adherence and follow-up, it may be acceptable to reduce the frequency of VL monitoring to 12-monthly.)

If the VL is > 50 copies/mL, then the patient should receive counselling, and interventions should be implemented to improve adherence. A repeat measurement of VL should then be done in 2–3 months.

We recommend a baseline VL for the following reasons:

- the 3-month VL can then be compared with the baseline VL to detect > 2 log10 drop, and if this has not occurred it allows early adherence intervention;
- it may guide NNRTI selection (RPV should not be used if VL > 100 000 copies/mL); and
- it confirms the diagnosis of HIV (antibody tests very rarely may give a false-positive result).

#### Interpreting viral load results

A VL > 50 copies/mL while receiving ART should be an indication for *urgent* action to improve adherence. A subsequent ART change must be considered if the patient meets the criteria for a switch to a second-line ART regimen at the subsequent 2–3-month follow-up VL measurement (see the section ‘Indications for changing antiretroviral therapy’).

VL monitoring is key to the success of ART. Decisions to change ART made on the basis of virological failure, rather than on clinical or immunological failure alone, have been shown to result in better patient outcomes.^28^ If the VL is undetectable, then the virus cannot mutate and develop resistance. A sustained VL of < 50 copies/mL is associated with the most durable benefit.

Isolated, detectable HIV VLs that are < 1000 copies/mL, which are followed by an undetectable VL, are termed ‘viral blips’ and alone are not a reason to change ART regimen.

### CD4+ counts

CD4+ counts should be performed every six months. In patients being monitored with VL measurements, if the CD4+ count at baseline was > 200 cells/μL or it increases above this threshold on ART, routine CD4+ testing can be stopped once the VL is suppressed and provided it remains suppressed, as it adds little to management. Data to support this have been summarised.^29^ However, if virological or clinical failure occurs, then a CD4+ count should be repeated, as cotrimoxazole (CTX) prophylaxis should be commenced if the count falls to < 200 cells/μL while receiving ART.

### Laboratory monitoring of antiretroviral therapy safety

Table 5 lists the blood tests and their frequency that we advise for monitoring of ART safety. The section ‘Antiretroviral therapy toxicity monitoring and management’ provides more details regarding the toxicities of ART and their management.

**TABLE 5:** Standard laboratory monitoring of patients once commenced on antiretroviral therapy.

Test	When		Comments
	Baseline	Ongoing	
VL†	Yes	3 months; 6 months and then 6-monthly	If VL undetectable for > 12 months, can reduce to 12-monthly
CD4 count†	Yes	6-monthly at virological or clinical failure	Can be stopped if CD4 > 200 and virologically suppressed
FBC†	Yes	Monthly for 3 months; at 6 months	For patients on AZT-containing regimens
ALT†	Yes	After initiation: 2, 4, 8 and 12 weeks	Only routinely monitored for patients on NVP-containing regimens and where the result is available quickly or the patient is easily contactable to come back if there is a problem
Creatinine clearance†	Yes	3 months; 6 months and then 6-monthly	Also at 1 month and 2 months in high risk patients. If symptoms of tubular wasting (e.g. muscle weakness) check potassium and phosphate levels
Total cholesterol and triglycerides (fasting)†	Not routinely	After 3 months on PI-containing regimen	If normal at 3 months, reassess only if other cardiovascular risk factors are present

† , This recommended routine monitoring ensures a standard level of care is given to patients on ART. However, it does not replace clinical judgement. These tests should also be carried out when clinically indicated, based on the discretion of the clinician.

VL, viral load; AZT, zidovudine; FBC, full blood count; ALT, alanine transaminase; NVP, nevirapine; PI, protease inhibitor.

## Defining antiretroviral therapy failure

In resource-limited settings where VLs are unavailable, the WHO has devised criteria for defining ART failure on the basis of CD4+ count responses or clinical disease progression. Studies have shown that switching ART regimens using these criteria results in a significant proportion of patients switching very late (with progressive accumulation of resistance mutations) and switching inappropriately (as the CD4+ count response may be poor in some patients, despite optimal virological suppression).^30^ We therefore advocate using the VL for making decisions regarding ART failure and the need to switch.

### Virological criteria for treatment success

Treatment success is defined by a decline in VL to < 50 copies/mL within six months of commencing ART, and sustained thereafter.

### Virological criteria for treatment failure

Treatment failure is defined by a confirmed VL of > 1000 copies/mL on two measurements taken 2–3 months apart. Several factors can influence the measurement of the VL. The decision to alter ART should, therefore, be based on the results of repeat testing after 2–3 months, following intensive adherence counselling. Inadequate patient adherence to the prescribed regimen remains the most common reason for treatment failure. Other important causes include: prior use of single-dose NVP for PMTCT; drug interactions that decrease ART concentrations; and transmitted drug resistance, which is currently increasing in the region (estimated to be around 5– 15%).^31,32^

### CD4+ response

Typically, the CD4+ count increases rapidly in the first month of ART, by approximately 75–100 cells/μL, with a more gradual rise thereafter (50–100 cells/μL/year).^33^ Most, but not all, patients achieve a CD4+ count > 500 cells/μL after several years of ART, provided that the VL remains suppressed.^34,35,36^ However, CD4+ responses are highly variable and may fail to increase despite virological suppression in about 10–20% of patients.^37,38^ Such patients have a delayed or absent CD4+ response to ART despite viral suppression, which is termed an ‘immunological discordant response to ART’. Certain studies suggest that older patients are at higher risk. There is no evidence that such patients benefit from a change in ART regimen; therefore, the same regimen should be continued. CTX prophylaxis should be continued if the CD4+ count remains < 200 cells/μL. There is evidence that the prognosis of such patients is worse than in those who have a CD4+ response, but better than that of patients experiencing both virological and immunological failure.^38^ If patients with an immunological discordant response to ART are clinically unwell, then TB or lymphoma should be considered as the cause of persistent CD4+ lymphopenia.

CD4+ counts may remain stable in the presence of incomplete viral suppression in patients receiving ART until the VL is high (approximately ≥ 10 000 copies/mL).^39^

## Indications for changing antiretroviral therapy

Individual ART drugs may be substituted in the event of toxicity (see the section ‘Antiretroviral therapy toxicity monitoring and management’), provided that the VL is suppressed or ART is initiated within the preceding six months. Changing the first-line ART regimen to a second-line regimen is a major step. The drugs used in second-line regimens are often not as well tolerated and are more expensive and generally require twice-daily dosing. For this reason, clinicians tend to switch to second-line ART after a prolonged period of virological failure, causing a progressive increase in the accumulation of resistance mutations. If the VL is detectable, it is essential to step up adherence interventions, as discussed above. If the patient is on an NNRTI-based first-line regimen, we advise a switch to a second-line regimen without undue delay when two VL measurements have been > 1000 copies/mL, preferably with the measurements taken 2–3 months apart, with at least four weeks of an intensified adherence intervention in between. In patients with low CD4+ counts (< 100 cells/μL), this process should be expedited. Some patients have persistently detectable VLs at low levels (200–1000 copies/mL). If patients have low-level viraemia (i.e. VL detectable but < 1000 copies/mL) for a prolonged period (> 1 year), or persistently low CD4+ counts (< 100 cells/μL) together with low-level viraemia despite adherence interventions, then they should be switched to second-line ART.

In patients who fulfil the criteria for virological failure on a dolutegravir-based regimen, there is currently an evidence gap with respect to data that inform the approach to switching to second-line ART. Because of the high barrier to resistance of DTG, it is likely that many such patients will not have resistance and will merely require improved adherence on the same first-line regimen. For that reason, we only recommend switching from first-line dolutegravir-based ART to second-line if a resistance test shows resistance. This advice may change as more data on the risks of resistance to dolutegravir-based ART in a programmatic setting become available. A similar approach should be applied for patients on a boosted PI first-line regimen.

## Second-line regimens

### Resistance testing for selecting second-line antiretroviral therapy

We do not recommend routinely performing a resistance test at first-line failure of NNRTI-based regimens. The EARNEST (Europe-Africa Research Network for Evaluation of Second-line Therapy) and SELECT (second-line ART in resource-limited settings) trials showed that without the use of a resistance test to decide which NRTIs to use in second-line therapy, virological outcomes were good and equivalent to a boosted PI + RAL regimen. In addition, in these trials those patients with more extensive NRTI resistance at first-line failure were those more likely to achieve virological suppression on second-line.^40,41^

A potential benefit of a resistance test is that it may be able to differentiate whether failure is because of complete or near complete non-adherence (when the resistance test shows no resistance mutations) and whether there is already established resistance (usually a consequence of sub-optimal adherence). Also, if the resistance test at first-line failure shows that there is no TDF resistance mutation, then TDF could be used to accompany 3TC (or FTC) and a ritonavir (RTV)-boosted PI in second-line.

When DTG is used in first-line ART, reports to date suggest that it is very unlikely that ART resistance will develop. There are inadequate data available for making a recommendation on resistance testing at DTG first-line failure, but similar recommendations as for second-line failure should be used until more data are available: patients with unsuppressed VL on a DTG-based first-line regimen should receive intensive adherence counselling and repeated VL measurements. Only if the VL remains detectable on several occasions despite this, should resistance testing be considered.

### Recommended second-line antiretroviral therapy regimen

We recommend a regimen of two NRTIs and a RTV-boosted (/r) PI. Boosting of PIs involves the addition of low-dose RTV, which inhibits PI metabolism, thereby boosting PI plasma concentration and prolonging half-life. We recommend against the use of unboosted PIs.

For patients who failed a first-line NNRTI + 2 NRTI regimen, we do not recommend the use of DTG with 2 NRTIs in second-line as there is currently insufficient evidence to support such a regimen. We await results of the DAWNING trial which will compare such a DTG-based versus LPV/r-based second-line regimen (https://clinicaltrials.gov/ct2/show/NCT02227238).

### Ritonavir-boosted protease inhibitor in the second-line regimen

Based on clinical trials demonstrating superior tolerability, we suggest that the preferred PI in second-line therapy should be ritonavir-boosted atazanavir (ATV) 300 mg/RTV 100 mg daily.^42,43^ The benefits of ATV/r over lopinavir (LPV)/r include that it is better tolerated in terms of gastrointestinal side effects, has a more favourable lipid profile and is taken once-daily. Drawbacks of ATV/r are: it cannot be used with rifampicin (RIF)-based TB treatment; and there are important drug interactions with drugs that reduce stomach acidity such as proton pump inhibitors. An alternative RTV-boosted PI rather than ATV/r should be used in the following situations:

- patients who do not tolerate ATV/r (e.g. cosmetically unacceptable jaundice): use LPV/r
- patients receiving RIF-based TB treatment: double-dose LPV/r should be used while receiving the TB treatment.

Other RTV-boosted PI options in second-line therapy are LPV/r and darunavir (DRV)/r. LPV is co-formulated with RTV in a heat-stable tablet (Aluvia). LPV/r is also an option with rifampicin when double-dosed. Darunavir/ritonavir (DRV/r) is taken twice-daily, currently, and is more costly. When the appropriate dose tablet becomes available, the 800/100 mg daily dose will be a feasible option in second-line ART, with fewer side effects than the twice-daily dosing. DRV/r cannot be co-prescribed with rifampicin.

Saquinavir (SQV) is not recommended.

Ritonavir capsules are no longer available and have been replaced with RTV heat-stable tablets.

### Selecting second-line dual nucleoside reverse transcriptase inhibitors

Because boosted PIs are robust drugs (i.e. resistance develops slowly) in PI-naive patients, it is very likely that virological suppression will be achieved with good adherence, even if the two NRTIs used in second-line therapy are partially compromised by NRTI resistance mutations (Tables 6 and 7). This is supported by findings of the EARNEST and SELECT trials, which showed good virological suppression rates of second-line LPV/r and NRTI regimens, even in patients with significant NRTI resistance.^40,41^ Certain NRTI combinations are contraindicated for toxicity reasons (e.g. d4T + didanosine [ddI], or TDF + ddI). A TDF + ABC combination is not recommended for second-line ART, as these agents share resistance mutations. NRTI combinations advised for second-line regimens include either AZT + 3TC, or TDF + 3TC (FTC can be substituted for 3TC), depending on the likely mutational profile selected during the patient’s first-line NRTI combination.

**TABLE 6:** Mutations selected by first-line nucleoside reverse transcriptase inhibitor combinations.

First-line NRTIs	NRTI mutations selected
3TC or FTC	Select for M184V, which compromises both 3TC and FTC and slightly impairs the activity of ABC and ddI, but increases susceptibility to AZT, d4T and TDF
AZT	Selects for TAMs, which may compromise all NRTIs†
d4T	Selects for TAMs, which may compromise all NRTIs. In a minority of patients, d4T may select for K65R, which compromises TDF, ABC and ddI, but increases susceptibility to AZT
TDF	Selects for K65R, which compromises TDF, ABC and ddI, but increases susceptibility to AZT. TDF also selects for K70E
ABC	Selects for L74V, which compromises ABC and ddI. May also select for K65R, which compromises TDF, ABC and ddI, but increases susceptibility to AZT. Selects for Y115F, which decreases its susceptibility

NRTI, nucleoside or nucleotide reverse transcriptase inhibitor; 3TC, lamivudine; FTC, emtricitabine; ABC, abacavir; ddI, didanosine; AZT = zidovudine; d4T, stavudine; TDF, tenofovir; TAMs, thymidine analogue mutations.

Note: These mutations accumulate with time – the longer the patient has virological failure, the more of these mutations are likely to be selected.

† , The presence of ≥ 3 TAMs, including M41L and L210W, confers intermediate-level to high-level TDF resistance.

**TABLE 7:** Choice of second-line nucleoside reverse transcriptase inhibitors in relation to first-line nucleoside transcriptase inhibitors used.

First-line NRTIs used	Second-line NRTI combination
AZT + 3TC	TDF + 3TC†
d4T +3TC	TDF + 3TC†
TDF + 3TC†	AZT + 3TC
ABC + 3TC	AZT + 3TC

NRTIs, nucleoside reverse transcriptase inhibitors; AZT, zidovudine; 3TC, lamivudine; TDF, tenofovir; d4T, stavudine; ABC, abacavir; FTC, emtricitabine.

† , 3TC is interchangeable with FTC as part of TDF/FTC combination tablet (FTC not available as single drug).

Even if 3TC (or FTC) was used in a failed first-line regimen and may, therefore, have selected for the M184V mutation, which confers resistance to the agent, 3TC (or FTC) should be reused in second-line therapy because of the capacity of the M184V mutation to partially restore susceptibility to AZT, d4T and TDF in the presence of thymidine analogue mutations (TAMs), and to partially restore susceptibility to TDF in the presence of the K65R mutation. The M184V mutation also reduces the replicative capacity of the virus.

In patients with baseline anaemia or who develop anaemia on second-line ART, AZT should be switched to ABC or TDF. In a minority of patients a second-line regimen of DTG plus a boosted PI may be considered (e.g. broad NRTI intolerance).

### Selecting second-line antiretroviral therapy in patients who received a first-line PI-regimen

If a patient was receiving a first-line combination of two NRTIs and a PI (boosted or unboosted), it is best to discuss the choice of second-line regimen with an experienced HIV clinician, and to perform a genotype resistance test. Second-line NNRTI + NRTI regimens are often not effective in such patients because of NRTI resistance mutations. The regimen choice is, therefore, best guided by resistance testing and DTG may have a role in such regimens.

## Third-line antiretroviral therapy regimens

**FIGURE 1:**
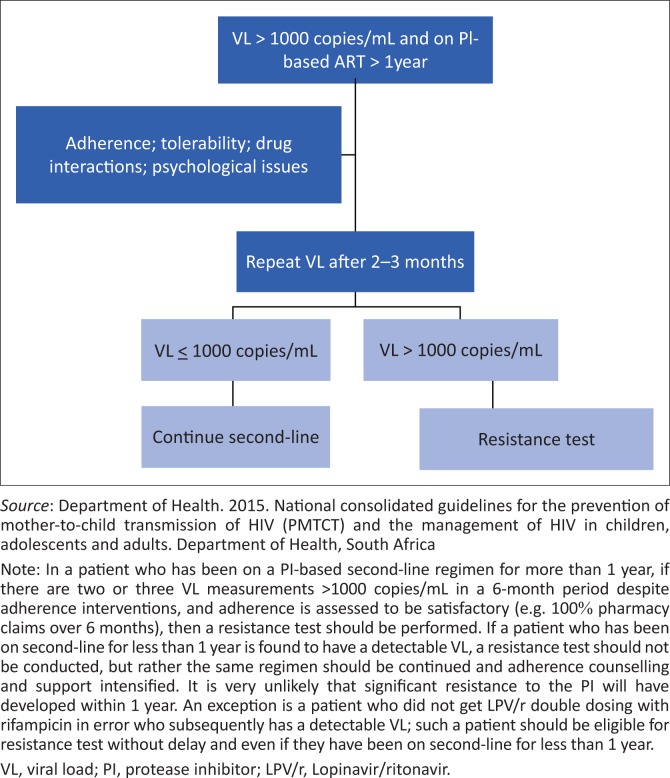
Indications for doing a resistance test on second-line antiretroviral therapy.

Third-line ART is used when a patient has experienced virological failure on drugs from the NRTI, NNRTI and PI classes, and has documented PI resistance. Before considering third-line therapy, adherence interventions should be intensified, and then adherence checked (e.g. check that pharmacy refills are all collected over a 6-month period). If there is still no viral suppression, then a resistance test should be performed to confirm the presence of resistance to the PI being used in second-line therapy. We advise doing this only after patients have been on a PI-based second-line regimen for a period of longer than one year, as the development of resistance earlier than this is very uncommon, unless there has been a medication error (e.g. giving standard dose LPV/r with rifampicin). The resistance test is expensive and the patient must be receiving the failing ART at the time, as ‘wild-type’ HIV is more fit and outgrows the resistant mutant population which therefore cannot be detected within some weeks after cessation of ART. However, third-line regimens are also expensive and are not justified if the patient does not have resistance necessitating such a switch. Currently, data show that most patients failing second-line regimens in the SA public sector have no PI mutations. In these patients, improved adherence is required rather than third-line regimens. See Figure 1.^44^

The decisions regarding treatment choices in third-line therapy are complex and need to be guided by resistance patterns found on resistance testing. It is essential that resistance tests are interpreted by an expert in conjunction with a full ART history.

A number of drugs are available for use in third-line ART: InSTIs (DTG and RAL), the newer PI DRV and newer NNRTIs (ETR and RPV)). These provide an opportunity for effective viral suppression with third-line therapy in the majority of patients, provided that adherence is optimal.^45,46^ Regimen choice should be individualised and an expert treater should always be consulted. A few guidelines regarding third-line ART regimens are:

- There is a need for specific adherence counselling in patients preparing to start third-line ART, with a frank discussion that this regimen is likely to be their last option for the foreseeable future.
- First-generation NNRTIs (NVP and EFV) have no place in third-line therapy as they do not impair viral fitness.
- A boosted PI with the broadest resistance profile should be selected (this is currently DRV). DRV must be used twice-daily in this context (600 mg 12-hourly with 100 mg RTV 12-hourly). LPV may be used if the drug is still active based on a resistance test (e.g. if the patient failed second-line ATV therapy).
- The addition of 3TC (or FTC) is recommended as the M184V mutation that it selects for impairs viral replication. Other NRTIs (the most active based on resistance testing) should also be added.
- Consideration of the addition of other drugs (e.g. DTG and ETR or RPV) will depend on the results of genotype resistance testing and cost issues. DTG is preferred because it belongs to an entirely new class with no risk of cross-resistance from prior ART exposure in first-line and second-line therapy, and has a higher barrier to resistance than RAL.^25^ Because most patients are not receiving an NNRTI at the time of failing second-line therapy when a genotype resistance test is typically performed, prior NNRTI mutations related to first-line NNRTI failure may be archived at this time. Therefore, it is difficult to be certain from this genotype whether ETR is compromised; however, data from SA suggest that the majority of patients who have failed NVP or EFV are still susceptible to ETR.^47^
- We advise against double RTV-boosted PIs.^48^
- If viral suppression is not achieved, then there is still benefit in continuing failing ART, because of the residual partial activity and ‘crippling’ effect of such ART. ‘Crippling’ describes the fact that mutant viruses often have less replicative capacity. Provided that the VL can be maintained at < 10 000 copies/mL, the CD4+ count will usually be maintained or even increase.^39^
- MVC (a CCR5 blocker) is a consideration in third-line therapy; however, it is currently extremely costly and can only be used after a tropism test shows that the patient’s circulating virus has sole tropism for the CCR5 coreceptor. We advise only considering this when there is intermediate-level or high-level resistance to all PIs, all NNRTIs and all NRTIs.

**FIGURE 2:**
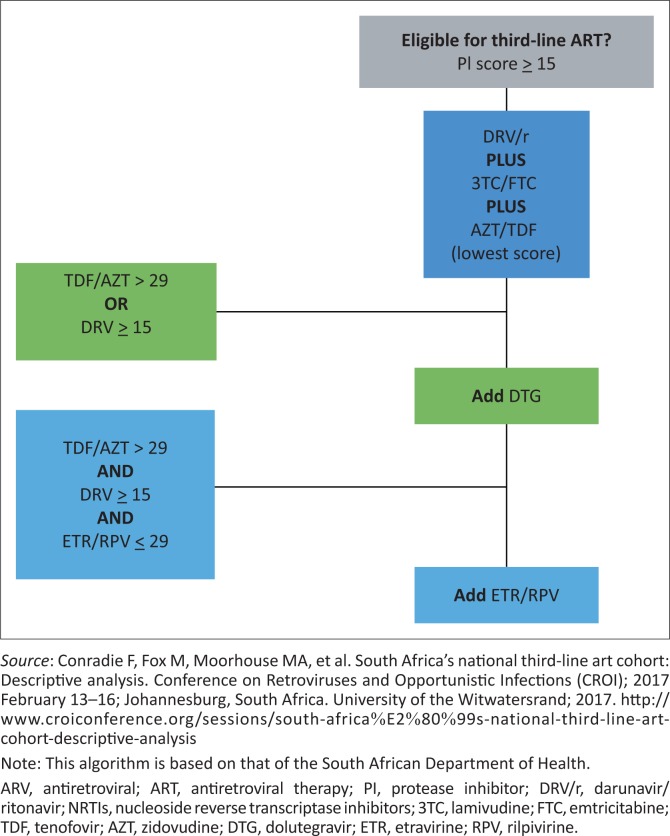
Algorithm for choosing drugs in third-line antiretroviral therapy regimen based on Stanford database score.

An algorithm approach to third-line ART can be used. Once a genotype resistance test has been performed, Stanford scores are used to determine which combination of ARVs is most appropriate for third-line regimen for the specific patient. Patients are eligible for third-line ART if they have demonstrated genotypic resistance to the PI which they are taking, as evidenced by a Stanford score ≥ 15, and should receive a DRV/r-based third-line regimen. In addition to DRV/r, all patients on a third-line regimen should receive two NRTIs, 3TC/FTC with either TDF or AZT, determined by which of TDF or AZT has the lower Stanford score. If there is resistance to DRV/r (score ≥ 15), or if there is intermediate-level or high-level resistance (score > 29) to the selected NRTI (TDF or AZT), add DTG to the regimen. If there is both DRV/r resistance and intermediate or high (score > 29) level resistance to the selected NRTI, then add ETR or RPV (whichever score is lower) in addition to DTG, unless both ETR and RPV score > 29. See Figure 2.^49^

The SAILING trial compared RAL versus DTG in ART-experienced patients with at least two class resistance who were integrase inhibitor-naïve. At week 48, DTG was superior to RAL (71% vs 64% achieved VL suppression < 50 copies/mL; *p* = 0.03). Significantly fewer patients developed treatment-emergent integrase inhibitor resistance in the DTG arm. We therefore no longer recommend the use of RAL in third-line unless DTG is not tolerated or there is a contraindication to using DTG. We also recommend that patients currently using RAL in third-line be switched to DTG, because of its higher barrier to resistance.^25^ If such patients have a suppressed VL then they can be switched to standard dose DTG (50 mg daily), but if they are not virologically suppressed we suggest a resistance test with integrase sequencing before switching. If there are InSTI mutations present that are associated with reduced susceptibility to DTG then the DTG dose should be 50 mg bd.

### Stopping antiretroviral therapy

Structured treatment interruptions are not advised as they have been shown to increase mortality (SMART study).^50^ Sometimes, patients need to stop therapy for reasons beyond the control of the patient and clinician. If life-threatening toxicity occurs (e.g. hepatitis with liver failure), all drugs should be stopped at once, but in most cases of toxicity, a continuation of ART should be attempted while switching the culprit drug(s) to an alternative. If stock-outs occur, it may also be necessary to stop ART. With PI and InSTI regimens, it is possible to stop all drugs simultaneously. With an NNRTI regimen, it is necessary to cover the tail with 5–7 days of two NRTIs (not needed if TDF/FTC are the NRTIs because of the long half-life of TDF and FTC).

We strongly advise against 3TC monotherapy ‘holding regimens’ in patients who have virological failure. Such regimens can be associated with a rapid fall in CD4+ count. The objective when prescribing ART should always be to provide a regimen that is most likely to achieve virological suppression.

## Patients who return after defaulting therapy

We recommend restarting the same regimen if patients return to care after defaulting therapy. A VL should preferably be performed before restarting. We then recommend that the VL is measured three months after restarting ART; switching to a second-line regimen should be considered if the VL is not < 1000 copies/mL at this point. In patients with multiple episodes of interruption, particularly beyond the first year of ART, many clinicians would consider switching to a second-line regimen, making the assumption that the multiple interruptions resulted in first-line resistance. Reasons for defaulting should be addressed and adherence support increased.

Hospitalisation with an AIDS-defining condition and a CD4+ count < 50 cells/μL represents another scenario in which a patient may be restarted immediately on second-line ART when returning to care after defaulting; the reason being that the patient is considered to be at high risk of mortality if restarted on a first-line therapy to which their virus may be resistant, and that they require a guaranteed effective ART regimen immediately. This decision should usually be taken by the clinicians at a hospital level.

Performing a resistance test after the patient has been off ART for longer than four weeks is of limited value, as many resistance mutations are overtaken by wild-type virus when ART is stopped.

## Drug interactions

There are many important drug interactions between ARV drugs and other medications, as well as between certain ARV drugs themselves. These interactions occur because of the metabolism of ARV drugs by cytochrome P450 in the liver and intestine, and the induction or inhibition by ARV drugs of this and other enzyme systems and drug transporters. Some of these drug interactions are discussed in these guidelines (e.g. the interaction between rifampicin and NNRTIs, PIs and InSTIs). The list of all potential drug interactions is, however, very long and beyond the scope of this document. Knowledge of drug interactions is constantly evolving. Clinicians are advised to consult package inserts of ARV drugs and concomitant medications to assess for drug interactions, in addition to the following websites, which provide up-to-date information on drug interactions and the actions required to account for them:

- University of Liverpool Drug Interactions Charts: http://www.hiv-druginteractions.org
- University of Cape Town Medicines Information Centre antiretroviral (ARV) interactions table: http://www.mic.uct.ac.za/?page_id=47

We advise that clinicians assess for potential drug interactions whenever patients start or switch to new ARV drugs or start new concomitant medications. This includes contraception. In addition, herbal medications may also have interactions with ARV drugs.

## Antiretroviral therapy in special populations

### Tuberculosis

The ARV regimen should be modified if necessary for compatibility with rifampicin. Rifampicin is a critical component of the TB regimen that substantially reduces the risk of relapse after completing TB treatment. EFV is the preferred NNRTI for use with rifampicin. NVP is an alternative in patients with contraindications for EFV (e.g. psychosis), but it carries a higher risk of hepatitis and virological failure when used with rifampicin. RPV and ETR cannot be used with rifampicin. DTG can also be used in patients on rifampicin, but a dose adjustment is required (Table 8).

**TABLE 8:** Antiretroviral therapy interactions with rifampicin and recommendations for co-administration.

Class	ART drug	Interaction	Dose of ART drug with rifampicin
NRTI	All in class	No significant pharmacokinetic interactions	No dose adjustment required.
NNRTI	EFV	Mild reduction in EFV concentrations. In some patients on TB treatment, EFV concentrations may increase	No dose adjustment required (600 mg *nocte*).
	NVP	Moderate reduction in NVP concentrations with increased risk of virological failure compared with EFV	Use standard dosing, but omit the lead-in dose phase and start 200 mg NVP 12-hourly.
	ETR and RPV	Marked reduction in concentrations	Do not prescribe concomitantly with rifampicin.
PI	LPV/r	LPV plasma concentrations significantly decreased	The preferable strategy is to double the dose of LPV/r to 800/200 mg 12-hourly. Alternatively, add 300 mg RTV 12-hourly to standard dose of two tablets of LPV/r 12-hourly. There is an increased risk of hepatotoxicity with these strategies. These dose adjustments can be made gradually over 1–2 weeks†.
	All other PIs	Marked reduction in PI concentrations	Do not prescribe concomitantly.
InSTI	RAL	Reduction in concentrations, but a clinical trial showed that standard dosing results in adequate virological suppression^51^	No dose adjustment required (i.e. RAL 400 mg 12-hourly).
	DTG	Significant reduction in concentrations	Dosing frequency increased to 50 mg 12-hourly.

ART, antiretroviral therapy; NRTI, nucleoside reverse transcriptase inhibitor; NNRTI, non-nucleoside reverse transcriptase inhibitor; EFV, efavirenz; NVP, nevirapine; ETR, etravirine; RPV, rilpivirine; PI, protease inhibitor; LPV, lopinavir; LPV/r, lopinavir/ritonavir; RTV, ritonavir; InSTI, integrase inhibitor (integrase strand transfer inhibitor); RAL, raltegravir; DTG, dolutegravir.

† , The double dosing regimen is preferred as it is better tolerated. Dose adjustments should be continued for 2 weeks after rifampicin is stopped.

The plasma concentrations of all boosted PIs are reduced to subtherapeutic ranges with rifampicin. Dose adjustment of LPV/r can overcome this induction (Table 8), but there is a risk of hepatotoxicity: patients require counselling and ALT should be monitored frequently.

An alternative approach is to replace rifampicin with rifabutin. However, rifabutin is not currently widely available at public sector TB clinics. Also, rifabutin is not co-formulated with other TB drugs, and the evidence base for rifabutin in the treatment of TB is much less substantial than that for rifampicin.^52^ There is also uncertainty regarding the optimal dose of rifabutin with boosted PIs; these guidelines recommend 150 mg daily (Table 9) for efficacy reasons but careful monitoring for toxicity is required (ALT, neutrophil count and visual symptoms at least monthly).^53^ Rifabutin may be considered in patients who are not tolerating co-treatment with LPV/r and rifampicin-based antitubercular therapy (patients unable to tolerate the increased LPV/r dose because of hepatotoxicity or gastrointestinal side effects) or in ART-experienced patients on an ART regimen that is not compatible with rifampicin (e.g. third-line ART with DRV/r). If rifabutin is unavailable and adjusted doses of LPV/r are poorly tolerated in patients receiving second-line ART, then DTG (50 mg 12-hourly) may be substituted for the PI. However, there is not good evidence regarding the robustness of DTG in second-line with compromised NRTIs, as there is for boosted PIs. Nevertheless, short-term use over six months is probably preferable to treating TB without rifampicin, which has a high risk of failure or relapse. ART and TB medication share many side effects (Table 10).

**TABLE 9:** Dosing of antiretroviral therapy drugs and rifabutin when prescribed concomitantly.

ART drug	ART dosage	Rifabutin dosage
EFV	No change	Increase to 450 mg/day
NVP	No change	300 mg/day
ATV or RTV-boosted PIs	No change	Decrease to 150 mg/day (monitor ALT, neutrophils and visual symptoms at least monthly)

ART, antiretroviral therapy; EFV, efavirenz; NVP, nevirapine; ATV, atazanavir; RTV, ritonavir; PIs, protease inhibitors; ALT, alanine transaminase.

**TABLE 10:** Shared side effects of antiretroviral therapy and tuberculosis treatment.

Side effects	ART	TB treatment
Nausea	AZT, ddI, PIs	Pyrazinamide, ethionamide
Hepatitis	NVP, EFV, PIs (NRTIs can cause steatohepatitis)	RIF, rifabutin, INH, pyrazinamide, bedaquiline and many second-line drugs, including quinolones
Peripheral neuropathy	d4T, ddI	INH, ethionamide, terizidone/cycloserine, linezolid
Renal impairment	TDF	Aminoglycosides, RIF (rare)
Rash	NVP, EFV, RAL	RIF, rifabutin, INH, pyrazinamide, ethambutol, streptomycin and many second-line drugs, including quinolones
Neuropsychiatric complications	EFV, DTG	Terizidone/cycloserine, quinolones, INH
Myelosuppression	AZT	Rifabutin and linezolid

ART, antiretroviral therapy; TB, tuberculosis; AZT, zidovudine; ddI, didanosine; PIs, protease inhibitors; NVP, nevirapine; EFV, efavirenz; NRTIs, nucleoside reverse transcriptase inhibitors; RIF, rifampicin; INH, isoniazid; d4T, stavudine; TDF, tenofovir; RAL, raltegravir; DTG, dolutegravir.

### Pregnancy

The aim of ART in pregnancy is both to prevent clinical complications of HIV in the mother and to decrease transmission to the child during pregnancy, delivery and breastfeeding.

#### Antiretroviral drugs in pregnancy

We recommend standard first-line, second-line and third-line regimens be used in pregnancy. Based on the accumulated evidence, we endorse the WHO guidance that EFV can be used in pregnancy and in women who intend to fall pregnant.^54^ Their guidance was based on a meta-analysis which found that the incidence of neural tube defects and all congenital abnormalities among women exposed to EFV in the first trimester was similar to that of the general population.^55^ The FDA category D classification of EFV should be discussed with women, explaining that this was based on animal studies; human cohort studies have not demonstrated an increased risk of congenital abnormalities, but that there is a background low risk of congenital abnormalities in all pregnancies, unrelated to drugs.

Studies have shown that total LPV concentrations are significantly reduced in pregnancy, but unbound LPV concentrations are not affected.^56^ Therefore, dose adjustment of LPV/r is not recommended in pregnancy. However, once-daily dosing of LPV/r should not be used in pregnancy. Similarly, concentrations of boosted ATV are reduced in pregnancy, but trough concentrations are adequate and dose adjustments are not recommended in pregnancy.^57^ An exception is in patients who are also receiving TDF, as TDF may reduce ATV concentrations: ATV/r dose should be increased to 400 mg/100 mg daily in such patients during the second and third trimester of pregnancy. Unboosted ATV is not recommended in pregnancy.

#### Recommendations for antiretroviral-naïve pregnant patients

It is beyond the scope of these adult ART guidelines to provide comprehensive guidance for the management of pregnant women. Key recommendations are included but providers are encouraged to refer to the complete PMTCT guidelines should a woman living with HIV be confirmed pregnant in order to optimise management.

All HIV-negative or status unknown pregnant women should be offered an HIV test at confirmation of pregnancy. If the test result is negative, regular retesting should be performed every three months throughout pregnancy and breastfeeding to detect any new infection. Seroconversion during pregnancy or breastfeeding is associated with a substantial risk of MTCT because of the very high maternal VL during acute infection.

The following is recommended:

- All HIV-infected pregnant women should be initiated on lifelong triple-drug ART.
- The aim should be to initiate all pregnant women on treatment on the same day as pregnancy confirmation. Before initiating ART, a full history and examination, including TB screen, should be conducted. Reasons to defer same-day initiation would include suspected or confirmed active TB, suspected or confirmed CM, suspected renal dysfunction or active psychosis.
- Women who cannot be initiated on TDF/FTC/EFV on the same day because of a drug-related contraindication (e.g. active psychosis; renal impairment) should be provided with an alternate three-drug regimen. Where an alternative three-drug regimen cannot be provided on the same day, provide AZT monotherapy and ensure that a three-drug regimen is started as soon as possible.
- Women who cannot be initiated on TDF/FTC/EFV on the same day because of a clinical contraindication (e.g. headache, where cryptococcal or TB meningitis are suspected) should be discussed with an expert to weigh up the benefit of starting ART for PMTCT versus the risk of IRIS for the mother.
- If a woman presents during labour and is newly diagnosed HIV-infected or was known HIV-infected but not yet on ART, start triple-drug ART immediately, no matter what stage of labour.
- Viral load should be checked at three months on therapy and then 6-monthly thereafter throughout pregnancy and breastfeeding.
- Refer to PMTCT guidelines for recommended regimens for the baby.
- A clear history of previous PMTCT exposure should be taken from all women who report they are ART-naïve. They may have been exposed to single-dose NVP, AZT or even triple therapy during previous pregnancies which was discontinued after delivery or cessation of breastfeeding according to previous guidelines. In this situation, the woman may not perceive herself to have been on ART but her risk of first-line failure may be increased. If she received triple therapy in a previous pregnancy or during breastfeeding, which was then discontinued because of a high CD4+ count, then she should be considered as re-initiating ART. This would necessitate that a baseline VL be taken on the day of pregnancy confirmation and repeated again after two months to ensure viral suppression is occurring on the first-line regimen. If viral suppression is not confirmed after two months, then treatment failure and non-adherence need to be considered and managed as per existing guidelines.

#### Recommendations for antiretroviral-experienced pregnant patients

In women currently taking ART, check VL on the day of pregnancy confirmation, regardless of when it was last performed. If the VL is suppressed then continue the current regimen and reinforce adherence messaging. If the VL is not suppressed, then provide intensive adherence counselling and repeat the VL in one month. If there has not been > 1 log drop in VL then switch patients on first-line to second-line ART. In patients on second-line regimen, if the VL is not suppressed, request an HIV drug resistance test to assess the need for a third-line regimen and continue with adherence support. In women who have previously stopped ART and returned to care when pregnant but not on ART, check the VL and restart the regimen they were taking previously with intensive adherence counselling. Then repeat the VL test in two months. If there has not been > 1 log drop in VL, then switch patients on first-line to second-line ART. In patients on second-line regimen, request a resistance test to assess their need for third-line ART.

If a woman presents in pregnancy and the history suggests previous evidence of virological failure on first-line ART or high likelihood thereof before they stopped ART, the provider should start the woman on second-line ART from the outset to maximise the chances of rapid viral suppression and avoiding the possibility of MTCT with resistant virus. Refer to PMTCT guidelines for recommended prophylaxis regimens for the baby.

#### Breastfeeding

Late MTCT during breastfeeding remains a considerable problem. All HIV-positive mothers who are breastfeeding should be maintained on triple ART with VL monitoring conducted every six months (if previously virally suppressed) throughout the breastfeeding period.

Women should be provided with breastfeeding support, where available, including counselling about the importance of exclusive breastfeeding during the first six months of life, followed by the addition of complementary foods up to 24 months of life, in combination with ongoing high levels of adherence to ART and regular VL monitoring.

HIV-negative women should also seek to breastfeed for 24 months and should access retesting regularly, at least 3-monthly, throughout this time. If an HIV-negative woman tests HIV-positive, or an HIV-positive woman is found to have a high VL (> 1000 copies/mL) during breastfeeding, then a discussion should be undertaken to assess the risks and benefits of continuing breastfeeding in relation to the nutritional benefits versus the increased risk of mother- to-child-transmission of HIV. In this situation, the baby should be tested immediately for HIV, as an HIV-positive infant requires urgent treatment and should continue breastfeeding.

If breastfeeding is to be discontinued, ensure that a safe, adequate alternative is available.

#### Postnatal period

Retention in care and ART adherence often decline in the postnatal period. Women who do not attend for their ART supply should be tracked, traced and re-engaged in care. If a woman is found to have low adherence or a treatment interruption is suspected or confirmed then a VL should be repeated, regardless of when last done, and the woman should be provided with support. She should be assessed for post-natal depression, gender-based violence, substance abuse and other reasons for low adherence.

#### Partner engagement

It is important to engage partners of pregnant women:

- Pregnancy indicates condomless sex. Encourage partner testing and engagement in care where necessary.
- Male involvement in antenatal and postnatal care has been shown to improve ART adherence and maternal and infant outcomes.
- Advise that the couple return to consistent condom use throughout the pregnancy to avoid increased risk of HIV transmission (if serodiscordant couple), reinfection (if concordant) and acquisition of new sexually transmitted infections (STIs) during the pregnancy, which can be associated with increased risk of miscarriage, preterm labour and intrauterine growth retardation.

### Renal failure

#### Antiretroviral drug dosages

Renal function is estimated either by the modified Cockgraft–Gault equation (Table 11) or the modification of diet in renal disease (MDRD) method, which most laboratories report as eGFR. The results of these formulae differ slightly, but either can be used for clinical management. For peritoneal dialysis, the dose given with a CrCl < 10 mL/min should be given daily. For haemodialysis, the dose given with a CrCl < 10 mL/min should be given daily.

**TABLE 11:** Antiretroviral drug dosage adjustments in the event of renal failure.

Drug	CrCl ‡§		Haemodialysis (dose after dialysis)	Peritoneal dialysis
	10 mL/min–50 mL/min	< 10 mL/min		
TDF	AVOID	AVOID	300 mg once weekly	Unknown
ABC	Unchanged	Unchanged	Unchanged	Unchanged
3TC	150 mg daily	50 mg daily†	50 mg first dose and thereafter 25 mg daily†	50 mg first dose and thereafter 25 mg daily†
AZT	Unchanged	300 mg daily	300 mg daily	300 mg daily
d4T	15 mg, 12-hourly	15 mg daily	15 mg daily	Unknown
ddI	> 60 kg body weight: 200 mg daily;	> 60 kg body weight: 125 mg daily;	> 60 kg body weight: 125 mg daily;	> 60 kg body weight: 125 mg daily;
	< 60 kg body weight: 150 mg daily	< 60 kg body weight: 75 mg daily	< 60 kg body weight: 75 mg daily	< 60 kg body weight: 75 mg daily
NNRTIs	Unchanged	Unchanged	Unchanged	Unchanged
PIs	Unchanged	Unchanged	Unchanged	Unchanged
InSTIs	Unchanged	Unchanged	Unchanged	Unchanged

*Source*: Please see the full reference list of the article, Meintjes G, Moorhouse MA, Carmona S, et al. Adult antiretroviral therapy guidelines 2017. S Afr J HIV Med. 2017;18(1), a776. https://doi.org/10.4102/sajhivmed.v18i1.776, for more information

CrCl, creatinine clearance; TDF, tenofovir; ABC, abacavir; 3TC, lamivudine; AZT, zidovudine; d4T, stavudine; ddI, didanosine; NNRTIs, non-nucleoside reverse transcriptase inhibitors; PIs, protease inhibitors; InSTIs, integrase strand transfer inhibitors; eGFR, estimated glomerular filtration rate; NRTIs, nucleoside reverse transcriptase inhibitors; MDRD, modification of diet in renal disease.

† , Some experts recommend that the lowest available tablet dose of 150 mg 3TC daily be used in patients with advanced renal disease (CrCl < 10 mL/min) and patients on dialysis so as to avoid having to use the liquid formulation of 3TC, and because of the favourable safety profile and lack of data to suggest 3TC dose-related toxicity.59 This is particularly relevant if the 3TC liquid formulation is unavailable or not tolerated; ‡, The modified Cockgraft–Gault equation: CrCl = (140 – age × ideal weight) ÷ serum creatinine. For women, multiply the total by 0.85; §, Many laboratories report the eGFR calculated by using a variation of the MDRD formula. This result can be used (in place of the calculated CrCl) to make decisions regarding the use of TDF and for modification of the dose of other NRTIs based on this table.

#### Antiretroviral drug choice and dosing in patients on chronic haemodialysis

Patients with HIV may develop end-stage renal failure requiring chronic haemodialysis owing to HIV-associated nephropathy or an HIV-unrelated cause. In patients on chronic haemodialysis, there are a number of important ART issues that arise. The NRTI class is eliminated through the kidneys, and thus doses of most NRTI drugs need to be adjusted in patients on dialysis (Table 11). Although TDF can be used in patients on chronic haemodialysis, dosing is once weekly, which can be difficult for patients to remember. AZT is generally avoided because of anaemia associated with renal failure. NNRTI drugs do not require dose adjustment.

We recommend the following first-line options for patients on chronic haemodialysis:

- ABC 600 mg daily
- 3TC 50 mg first dose and thereafter 25 mg daily (on the days when haemodialysis is performed, the dose should be given after the haemodialysis session)
- EFV 600 mg nocte.

Atazanavir concentrations are reduced in patients on haemodialysis to a greater degree than LPV, and thus ATV is preferably not used in treatment-experienced patients. LPV/r should be used with twice-daily dosing in patients on haemodialysis. In patients on haemodialysis, we recommend an LPV/r-based second-line regimen with two NRTIs in the regimen (selected after consideration of issues related to NRTIs discussed above). DRV/r and InSTIs may be used at standard dosages. It is suggested that patients on chronic haemodialysis (who generally receive ongoing medical care in a dialysis unit) are reviewed by a clinician experienced in ART management at least 6-monthly, to monitor treatment efficacy and side effects and to adjust the regimen when needed.

Daily dosages or the evening doses of a twice-daily ART regimen on the day of haemodialysis should be given *after* the haemodialysis session to prevent the drug from being dialysed out.

#### Antiretroviral therapy in patients with acute kidney injury

In patients with acute kidney injury (AKI), dosages of NRTI drugs should be adjusted based on estimated CrCl calculation (Table 11).^58,59,60^ TDF should be interrupted even if it is not thought to be the cause of the AKI. Once there is clear evidence that renal function is improving (creatinine on downward trend), NRTI dosages should be readjusted to standard dosages to avoid underdosing. In patients with AKI who are not yet receiving ART, initiation is preferably deferred until AKI has resolved. See the section ‘Nephrotoxicity’ for discussion of ART nephrotoxicity.

### Antiretroviral therapy dosages in liver impairment

Unlike in renal impairment, there is no blood test that can accurately quantify liver impairment. Child-Pugh class C may require dose adjustment for the relevant ART drugs listed in Table 12. In general, the combination of TDF with 3TC (or FTC) and DTG or RAL (or EFV, which can be hepatotoxic) is regarded as the least hepatotoxic.

**TABLE 12:** Prescribing antiretrovirals in liver impairment.

Class	Drug	Prescribing notes
NRTIs	TDF	In patients with chronic hepatitis B, there is a risk of rebound hepatitis when TDF is discontinued
	FTC	In patients with chronic hepatitis B, there is a risk of rebound hepatitis when FTC is discontinued or if hepatitis B resistance to FTC develops
	3TC	In patients with chronic hepatitis B, there is a risk of rebound hepatitis when 3TC is discontinued or if hepatitis B resistance to 3TC develops
	AZT	Decrease dose by 50% or double dosage interval if significant liver disease
	ABC	Reduce adult dose to 200 mg bd for significant liver impairment. Contraindicated in severe hepatic impairment
	d4T	Use with caution
	ddI	Use with caution: reports implicate use as a risk factor for the development of hepatic decompensation in patients being treated for cirrhosis because of hepatitis C
NNRTIs	EFV	Caution should be exercised in administering EFV to patients with liver disease; therapeutic drug monitoring should be done if available
	NVP	Avoid if significant hepatic impairment or active hepatitis B or C
	ETR	Use with caution in severe liver disease (Child-Pugh Class C) as dosage recommendation has not been established
	RPV	Use with caution in severe liver disease (Child-Pugh Class C) as dosage recommendation has not been established
PIs	ATV	Avoid in severe hepatic impairment
	LPV/r	LPV is highly metabolised in the liver and concentrations may be increased in patients with hepatic impairment; therapeutic drug monitoring should be done if available
	DRV	Use with caution or avoid if significant liver disease
	SQV	Avoid: there have been reports of worsening liver disease and development of portal hypertension after starting SQV in patients with severe liver disease
InSTI	RAL	No dosage adjustment necessary in mild-to-moderate hepatic insufficiency
	DTG	Not recommended in severe liver disease (Child-Pugh Class C)
CCR5 blocker	MVC	Concentrations likely to be increased with hepatic impairment

NRTIs, nucleoside reverse transcriptase inhibitors; TDF, tenofovir; FTC, emtricitabine; 3TC, lamivudine; AZT, zidovudine; ABC, abacavir; bd, twice-daily; d4T, stavudine; ddI, didanosine; NNRTIs, non-nucleoside reverse transcriptase inhibitors; EFV, efavirenz; NVP, nevirapine; ETR, etravirine; RPV, rilpivirine; PIs, protease inhibitors; ATV, atazanavir; LPV/r, lopinavir/ritonavir; DRV, darunavir; SQV, saquinavir; InSTI, integrase inhibitor (integrase strand transfer inhibitor); RAL, raltegravir; DTG, dolutegravir; CCR5, C-C chemokine receptor type 5; MVC, maraviroc.

### Hepatitis B co-infection

Hepatitis B is a common co-infection with HIV in southern Africa, with significant implications for progression to cirrhosis, as well as for treatment options. Clinicians are encouraged to support current efforts in the region to vaccinate all children for hepatitis B, and to extend coverage to eligible adults. Access to vaccination, laboratory resources and treatment options are limited to some extent in southern African countries, and the recommendations below should each be considered in the light of the local context.

All HIV-infected patients should be screened for active hepatitis B (limiting screening to those with liver function abnormalities will miss many cases, as liver enzymes are often normal in hepatitis B infection). HBsAg is an appropriate screening test. Hepatitis B VL correlates with disease progression and may be used to monitor antihepatitis B therapy, but it is expensive and availability is limited.

Hepatitis B/HIV co-infection is associated with:

- an increased risk of chronic liver disease
- a higher hepatitis B VL
- diminished responses to hepatitis B vaccine
- poorer responses to interferon-alpha treatment
- an increased incidence of drug-induced hepatotoxicity (particularly with NVP)
- a flare of hepatitis within three months of commencing ART (because of hepatitis B-IRIS, which is difficult to differentiate from drug hepatotoxicity).

Drugs directed against hepatitis B that have no or minimal anti-HIV activity (e.g. entecavir and telbivudine) are largely unavailable or extremely expensive in our region. For practical purposes, the only available therapy is to use ART drugs that also have antihepatitis B activity (TDF + 3TC/FTC). As with HIV, these drugs suppress hepatitis B, but do not eradicate it. Effective treatment prevents or slows progression to cirrhosis. For all HIV-infected HBsAg-positive patients the ART regimen should include TDF and 3TC (or FTC). Using 3TC without including TDF leads to hepatitis B resistance in 80–90% of patients after five years of treatment. If a patient meets the criteria for switching to a second-line ART regimen (to treat HIV), this combination (TDF + 3TC/FTC) should be continued to suppress hepatitis B infection, as interruption of TDF or FTC/3TC has been associated with flares of life-threatening hepatitis. The second-line ART regimen should be shaped around these two drugs. NVP should be avoided in patients with hepatitis B co-infection.

In patients with hepatitis B and renal dysfunction, the use of TDF may be considered with dosing frequency adjustment based on CrCl (see package insert) and more frequent creatinine monitoring. If renal dysfunction is severe or renal function deteriorates with TDF, then 3TC monotherapy (with or without pegylated interferon-alpha, which is very costly) or other drugs with antihepatitis B activity should be considered.

### Malaria

There are several drug interactions between antimalarials and ART drugs: Artemether-lumefantrine (Coartem) can safely be administered with NVP. EFV significantly lowers the concentrations of artemether (and its active metabolite) and lumefantrine, which is likely to increase the risk of failure of antimalarial therapy. There is no clear guideline on how to overcome this interaction, but some experts recommend repeating the 3-day course of artemether-lumefantrine (i.e. treat for six days). Boosted PIs dramatically increase the plasma concentrations of lumefantrine, but a dose reduction is not recommended, as the toxicity threshold of lumefantrine seems to be high. Close monitoring for toxicity is recommended when coadministering artemether-lumefantrine with ART.

Quinine concentrations are significantly decreased by LPV/r, probably owing to induction of metabolism by RTV. It is likely that quinine concentrations will also be reduced by EFV and NVP; therefore, quinine should be avoided in patients receiving PIs or NNRTIs. Patients with severe malaria should receive artesunate, if this is available, and those with milder malaria should be treated with artemether-lumefantrine.

Among drugs used for chemoprophylaxis, there are no clinically significant pharmacokinetic interactions between ARVs and mefloquine or doxycycline. However, mefloquine and EFV both cause frequent neuropsychiatric side effects; therefore, doxycycline is the preferred chemoprophylactic agent for patients receiving EFV.

There are several interactions with atovaquone-proguanil (Malanil). Atovaquone concentrations are reduced by PIs and EFV. It is also likely that NVP decreases atovaquone concentrations. Proguanil concentrations are also reduced by PIs and EFV. Use of atovaquone-proguanil is, therefore, best avoided in patients receiving PIs or NNRTIs.

No significant drug interactions are predicted between InSTIs and antimalarial drugs.

## Antiretroviral therapy toxicity monitoring and management

Currently recommended ART is generally well tolerated. Many adverse drug reactions are mild and occur only in the first few weeks of therapy. If toxicity does not resolve or is severe, then the offending drug should be substituted as indicated below. It is important to ensure that the VL is suppressed before substituting a single drug for toxicity; otherwise, resistance may develop to the new drug, consequently compromising future regimens. However, single drug substitutions can be done in the first few months of ART without measuring the VL, as the VL may take up to six months to suppress.

It is rarely necessary to stop the entire ART regimen because of toxicity. It is advised to switch only the culprit drug(s) and continue the rest of the ART regimen. In certain life-threatening situations (e.g. hepatitis with liver failure), it may be necessary to cease use of all ARVs.

### Haematological toxicity

Cytopenias occur commonly in HIV infection without exposure to ART. Patients receiving AZT, d4T or CTX may experience abnormalities in their FBCs. Significant bone marrow toxicity from CTX generally only occurs with high doses used for treating OIs. Patients receiving prophylactic CTX rarely develop isolated neutropenia. Hb and neutrophil monitoring is necessary with AZT; this should be performed monthly for three months, then after six months of therapy and thereafter if clinically indicated (it is unusual to see haematological toxicity developing after six months). The main problem arising from AZT use is anaemia and neutropenia; platelet counts generally rise with use of the drug. Management guidelines are provided in Table 13. Macrocytosis is usual with d4T and AZT therapy; there is no need to measure vitamin B12 and folate concentrations unless there are other indications that these may be deficient.

**TABLE 13:** Guidelines for managing haematological toxicity (mainly zidovudine-induced).

Hb (g/dL)	Hb low but > 8: Monitor weekly	< 8: Switch from AZT	
Neutrophils (× 109/L)	1.0–1.5: Repeat 4 weeks	0.75–0.99: Repeat 2 weeks Consider switching from AZT	< 0.75: Switch from AZT

AZT, zidovudine; Hb, haemoglobin.

Pure red cell aplasia, which presents with severe anaemia and low reticulocyte production index, has rarely been associated with 3TC and FTC. A bone marrow examination should be performed to confirm the condition. Parvovirus B19 infection should be excluded (a polymerase chain reaction [PCR] test should be requested on blood sent in an ethylenediaminetetra-acetic acid [EDTA] tube).

### Hepatotoxicity

Alanine transaminase should be performed at ART initiation and repeat ALT testing is indicated in patients who develop symptoms or signs suggestive of hepatitis. All ART classes have been associated with hepatotoxicity – most commonly NNRTIs. NRTIs very rarely present with acute hepatitis. Mild ALT elevations occur very commonly and usually transiently with many drugs in general. ALT elevations > 5× the upper limit of normal (ULN) are significant in the absence of symptoms. In the presence of symptoms of hepatitis, ALT elevations > 2.5× ULN are significant. Management guidelines are provided in Table 14.

**TABLE 14:** Guidelines for managing hepatotoxicity: Elevation.

ULN	< 2.5 × ULN	2.5–5 × ULN	> 5 × ULN
ALT	Repeat at 1–2 weeks	Repeat at 1 week	Discontinue relevant drug(s)
Bilirubin	Repeat at 1 week	Discontinue relevant drug(s)	Discontinue relevant drug(s)

Note: Any elevations with symptoms of hepatitis (nausea, vomiting, right-upper-quadrant pain) should be regarded as an indication to stop the relevant drugs.

ALT, alanine transaminase; ULN, upper limit of normal.

Nevirapine is the ARV drug most frequently associated with drug-induced liver injury (DILI) and most cases occur in the first three months of starting the drug. Ideally, in patients starting NVP, ALT should be monitored at 2, 4, 8 and 12 weeks after initiation. If monitoring is performed, a system should be in place to obtain the result and contact the patient. Routine ALT monitoring makes little sense in settings where the result will only be available when the patient is seen in 2–4 weeks, or where the patient cannot be contacted. It is essential to educate all patients starting NVP about the symptoms of hepatitis (nausea, vomiting, anorexia, malaise, jaundice and right-upper-quadrant pain) and drug rash, which is frequently associated with hepatitis. Hepatitis often follows the rash after about 10 days. If such symptoms develop, ALT should be determined urgently.

Hepatotoxic drugs should be discontinued at high levels of LFT abnormality or at lower levels if any symptoms of hepatitis appear and an alternative ARV drug substituted. Rechallenge may be considered in selected cases; a specialist should be consulted. If hepatitis occurs together with a rash or fever, or with other systemic involvement, then rechallenge with NNRTIs, ABC or CTX should not be attempted.

Prolonged use of NRTIs, especially d4T and ddI, may cause fatty liver. Typically, ALT concentration is more significantly elevated than AST, and the concentrations of canalicular enzymes (gamma-glutamyl transferase [GGT] and alkaline phosphatase [ALP]) are more elevated than those of the transaminases. Non-tender hepatomegaly may be present. Ultrasound or computed tomography (CT) imaging may show decreased hepatic density. The condition is not benign and fibrosis has been reported with long-term ddI use. Patients should be advised to avoid alcohol and switched to alternative dugs with lower potential for causing fatty liver.

In patients with severe hepatitis or jaundice, the international normalised ratio (INR) and serum glucose should be assessed, as well as features of hepatic encephalopathy (i.e. features of hepatic failure).

If the concentration of canalicular enzymes is more significantly elevated than that of ALT, or if conjugated bilirubin is elevated, an ultrasound of the liver should be conducted to exclude biliary obstruction.

Isolated unconjugated hyperbilirubinaemia (drug-induced Gilbert’s syndrome) is associated with ATV. In this case, all other LFTs are normal and the patient has no other symptoms of hepatitis. Although this is a benign condition (it does not reflect liver injury, but isolated competitive inhibition of the enzyme in the liver which conjugates bilirubin), it is often cosmetically unacceptable to patients requiring a switch from ATV to an alternative.

While EFV has been recognised to be an infrequent cause of DILI since it first became available, recently a novel pattern of DILI associated with EFV has been recognised.^61^ Among these patients, many were found to have a particularly severe pattern of liver injury at liver biopsy (termed ‘submassive necrosis’, and associated with severe jaundice and raised INR) and overall mortality was 11%. While severe EFV-related DILI is likely to be uncommon, clinicians should be aware of the features observed:

- The diagnosis of DILI was generally made after a longer duration on EFV than what is seen with DILI related to NVP or TB medication: around 3–6 months on EFV.
- The DILI was not associated with features of hypersensitivity (e.g. drug rash) and often the first symptom was jaundice rather than abdominal symptoms.
- Once EFV was stopped it typically took several months for liver function tests to normalise. Median resolution took more than six months.

We do not advise routine liver function test monitoring in patients on EFV, as there is no evidence that this would lead to earlier detection of this DILI or improve outcomes. However, those managing patients on EFV should monitor for symptoms and signs of hepatitis (nausea, vomiting, right-sided abdominal pain or jaundice). If these occur, an ALT should be requested and the patient examined for jaundice. EFV should be switched to an alternative (e.g. DTG) and the patient managed appropriately for DILI if there are hepatitis symptoms with ALT > 120, or there is jaundice.

Many other drugs can cause hepatotoxicity, notably antituberculous agents (including prophylactic isoniazid) and azoles. CTX is an uncommon cause of hepatitis, often as part of a systemic hypersensitivity reaction.

Recommendations for the management of DILI in patients receiving TB treatment have been published by the Society in 2013.^62^

### Hyperlactataemia and lactic acidosis

- This side effect has become less common with fewer patients starting ART with d4T and ddI. It can also occur occasionally with AZT.
- Lactic acidosis is a serious, rare, potentially fatal side effect of NRTIs, most commonly associated with d4T, particularly when combined with ddI. Symptomatic hyperlactataemia without acidosis is more common, but seldom seen with the safer NRTIs that are currently recommended.
- Symptoms are nonspecific and include nausea and vomiting, abdominal pain, dyspnoea, fatigue and weight loss.
- Risk factors for hyperlactataemia include female gender, obesity and use of NRTIs for more than six months.
- A raised lactate of > 5 mmol/L together with metabolic acidosis confirms the diagnosis of lactic acidosis. Low serum bicarbonate (< 20 mmol/L) is the most sensitive marker of acidosis.

The management of symptomatic hyperlactataemia depends on lactate and bicarbonate concentrations:

- Lactate < 5 mmol/L and bicarbonate > 20 mmol/L: NRTIs should be switched to agents less frequently associated with hyperlactataemia: TDF/ABC + FTC/3TC. Symptoms and serial lactate should be monitored for several months (lactate levels decrease slowly over weeks).
- Lactate > 5 mmol/L and bicarbonate > 15 mmol/L: Patient should be admitted and NRTIs should be discontinued. If the patient is on an NNRTI regimen, then a boosted PI should be added. If the patient has already failed an NNRTI and is on a boosted PI, then DTG should be added, if available, or the patient should be continued on the boosted PI only. When lactate has normalised, the patient should be switched to TDF/ABC + 3TC/FTC, as above.
- Lactate > 5 mmol/L and bicarbonate < 15 mmol/L: The patient should be admitted, preferably to an ICU, and NRTIs should be discontinued. If the patient is on an NNRTI regimen, a boosted PI should be added. If the patient has already failed an NNRTI regimen and is receiving a boosted PI, then DTG should be added, if available, or the patient should be continued on a boosted PI only. Bicarbonate replacement is controversial, but most experts would use this strategy to partially correct severe acidosis. Broad-spectrum antibiotics are recommended as sepsis can mimic NRTI-induced lactic acidosis (this can be discontinued if procalcitonin is normal). On recovery, all NRTIs should be avoided in future regimens.

### Dyslipidaemia

PIs can cause hypertriglyceridaemia and elevated low-density lipoprotein (LDL) cholesterol. Boosted ATV and once-daily boosted DRV (800 mg DRV/100 mg RTV once-daily) are associated with less severe dyslipidaemia than other boosted PIs; d4T and AZT can cause mild hypertriglyceridaemia, and EFV can cause elevated total cholesterol and mild hypertriglyceridaemia.

We suggest routinely assessing lipids after three months on a PI-regimen. If normal at this stage, reassessment should be performed only in those with cardiovascular (CVS) risk factors.

Diet and lifestyle modification should always be advised. Diet is more effective for controlling hypertriglyceridaemia than hypercholesterolaemia. Other CVS risk factors should be addressed. Clinicians should consider and investigate secondary causes of hypertriglyceridaemia and hypercholesterolaemia (e.g. diabetes, nephrotic syndrome, alcohol abuse and hypothyroidism).

If patients on LPV/r develop significant dyslipidaemia, then they should be switched to ATV/r, if possible, rather than adding lipid-lowering therapy. However, lipid-lowering therapy is indicated in patients with persistent elevations despite switching to ATV/r. Switching the PI to DTG is another option, because DTG has a more favourable lipid profile than PIs. However, DTG should only be used in a regimen in which at least one other ARV drug is known to be fully active. In patients with hyperlipidaemia on EFV, switch EFV to RPV.

Marked hypertriglyceridaemia (> 10 mmol/L) can cause pancreatitis and requires urgent treatment with diet modification (restrict total triglyceride intake to < 30 g/day), fibrates and switching LPV/r to ATV/r or DTG (fibrates can be stopped after one month, followed by reassessment within 4–6 weeks).

Indications for statin therapy in HIV-positive patients should be the same as in HIV-negative patients, using the Framingham heart disease risk score. As a general rule, in young patients with isolated elevated cholesterol but no other CVS risk factors, a threshold of total cholesterol > 7.5 mmol/L (or LDL cholesterol > 5.0 mmol/L) should be used for initiating statin therapy, and the patient should be referred to a lipid clinic for investigation if feasible. In patients with CVS risk factors (e.g. smoking, diabetes, hypertension) decisions should be made using the Framingham heart disease risk score (https://www.mdcalc.com/framingham-coronary-heart-disease-risk-score). All patients with established atherosclerotic disease (coronary, cerebral or peripheral), all patients with familial hypercholesterolaemia and all patients with type 2 diabetes aged > 40 years should be started on statin treatment.

Many statins have interactions with PIs that can lead to potentially toxic statin concentrations, with the exception of pravastatin and fluvastatin. Atorvastatin concentrations are significantly raised by PIs, but low doses (maximum 10 mg daily) can be used with monitoring for symptoms of myalgia. Lovastatin and simvastatin should not be coadministered with PIs, as their concentrations are dramatically increased and severe rhabdomyolysis has been reported. We also advise against the use of rosuvastatin with PIs because of a complex drug–drug interaction: PIs increase the plasma concentrations of rosuvastatin while reducing their efficacy in the liver.

### Lipodystrophy

Lipodystrophy can present with fat accumulation (visceral obesity, breast enlargement, ‘buffalo hump’ or lipomata) or subcutaneous fat loss (lipoatrophy, most noticeable in the face, limbs and buttocks), or with both forms of lipodystrophy.

The thymidine analogue NRTIs (AZT and especially d4T) are associated with fat loss. Lipoatrophy improves when d4T/AZT are substituted with TDF or ABC, but resolution is very slow and often incomplete; therefore, it is important to recognise lipoatrophy early or, better still, to use NRTIs that are not associated with the condition.

Previously, PIs were thought to be the cause of lipohypertrophy. However, more recent studies have shown that all ART classes are associated with fat gain to the same extent. Furthermore, longitudinal studies comparing HIV-negative people with HIV-positive people on long-term ART have demonstrated that the extent and distribution of fat gain are similar. A systematic review of RCTs concerning switching ART for fat accumulation failed to show any benefit.^63^ These data indicate that fat gain is a consequence of treating HIV rather than a drug-specific side effect. Telling the patient that the ART drugs are causing fat gain is not only incorrect, but may result in the patient discontinuing ART. The appearance of the fat gain is particularly unsightly when accompanied by lipoatrophy in the face, limbs and buttock.

There is no evidence to support the switching of ART drugs in patients with fat accumulation. Healthy diet and exercise should be advocated, as in the HIV-negative population. Surgery should be considered in selected cases with focal fat gain (e.g. those with prominent ‘buffalo humps’). Metformin modestly reduces weight and improves insulin resistance in patients with the metabolic syndrome or isolated dysglycaemia.

Visceral fat accumulation is associated with insulin resistance and dyslipidaemia. Other CVS risk factors should be addressed.

### Hypersensitivity

Rash with NNRTIs is common (more severe and frequent with NVP) in the first six weeks of therapy. If the rash is accompanied by systemic features (e.g. fever, elevated ALT or hepatitis), mucosal involvement or blistering, then the NNRTI should be discontinued immediately and rechallenge must not be performed as these are features of life-threatening reactions. If the rash is mild and occurs without these features, then the NNRTI can be continued and the rash can be treated symptomatically with antihistamines, and possibly topical steroids. Systemic steroids should not be used.

- In patients who develop mild rashes during the low-dose NVP lead-in phase (200 mg daily), the dosage must not be increased to 200 mg 12-hourly until the reaction has resolved. This ‘treat-through’ approach is only acceptable if the patient can be observed carefully, otherwise, NVP should be substituted.
- There is a possible cross-reaction between NVP and EFV, although most studies report no evidence of this. It is acceptable to substitute EFV for NVP in the event of hypersensitivity, unless the reaction was severe. In addition, if there was a severe reaction to NVP or EFV we do not recommend switching to RPV or ETR (rather use DTG or PI). There are hardly any data on substituting NVP for EFV in the event of hypersensitivity; therefore, this substitution is not recommended.
- ABC hypersensitivity is primarily a systemic reaction occurring within the first eight weeks of therapy in ~3% of cases. Fatalities may occur on rechallenge. ABC must be discontinued and never reintroduced. The manifestations of hypersensitivity include fever, rash, fatigue and abdominal or respiratory symptoms. If there is any doubt concerning the diagnosis (e.g. if the patient has a cough with fever), then the patient should be admitted for observation of the next dose: symptoms progress if hypersensitivity is present. The hypersensitivity reaction has been shown to occur on a genetic basis, being very strongly associated with the HLA-B*5701 allele, which is very uncommon in Africans. It is thus less frequent in patients of African descent. If testing is affordable and available, this allele should be excluded prior to using ABC.
- Other ARV drugs, notably RAL and DRV, can occasionally cause hypersensitivity rashes, including life-threatening rashes.
- CTX is a common cause of cutaneous and systemic hypersensitivity reactions, indistinguishable from hypersensitivity reactions to ART drugs. CTX should be interrupted when treating mild suspected NNRTI cutaneous hypersensitivity rashes, and permanently discontinued if severe hypersensitivity reactions occur. If being given for secondary prophylaxis an alternative should be substituted.

### Nephrotoxicity

In a minority of patients, TDF may cause a tubular wasting syndrome (including wasting of phosphate and potassium). If patients receiving TDF develop muscle weakness or other muscle symptoms, then potassium and phosphate levels must be assessed. TDF can also cause acute renal failure, but this is uncommon. TDF should be switched to ABC (or an alternative NRTI) immediately in patients with acute renal failure as it may exacerbate injury even if it is not the primary cause. Consider recommencing TDF with careful monitoring when the creatinine is normal only if an alternative cause of renal failure is established and TDF was considered not to have contributed.

We recommend that the CrCl should be estimated before commencing TDF, which should not be used if the eGFR or CrCl is < 50 mL/min. For patients receiving TDF, creatinine should be monitored at three months, six months and then 6-monthly thereafter. In high risk patients (particularly those with coexistent hypertension or diabetes), creatinine should also be checked at one and two months. Long-term use of TDF together with other nephrotoxic agents (e.g. aminoglycosides or non-steroidal anti-inflammatory agents [NSAIDs]) should be avoided. Where TDF is avoided because CrCl is < 50 mL/min at baseline, it may be possible to switch to TDF at a later point if renal function improves. This is often the case where patients had diarrhoea or other OIs at the time of ART initiation.

### Neuropsychiatric toxicity

Zidovudine and raltegravir frequently cause headaches when started, but this usually resolves. EFV frequently causes neuropsychiatric effects in the first few weeks of therapy, typically presenting with insomnia, vivid dreams and dizziness. Both dysphoria and euphoria may occur. Fortunately, these features subside in the majority of patients within the first 4–6 weeks. Psychosis may occasionally occur. If the neuropsychiatric effects of EFV are not tolerated, then the patient should be switched to RPV, DTG, lower-dose EFV or NVP (if the CD4+ count is < 250 cells/μL in women or < 400 cells/μL in men; if virologically suppressed, then EFV can be switched to NVP at higher CD4+ counts as this is not associated with increased risk of rash-associated hepatitis), 400 mg daily. Patients starting EFV should be warned about these symptoms, and reassured that they resolve in most patients continuing the drug, but if not, that an alternative can be substituted. DTG may also cause insomnia, headache and neuropsychiatric side effects. RAL has been associated with similar CNS side effects.

### Dysglycaemia

The older PIs, notably IDV, may cause diabetes. However, the newer PIs (ATV, DRV and LPV) do not. EFV, AZT and d4T are associated with small increased risks of dysglycaemia. Visceral fat gain, which occurs to a similar extent with all ART classes, is associated with insulin resistance. Blood glucose should be assessed serially in these patients as part of a CVS risk assessment.

### Gynaecomastia

Gynaecomastia, which is a benign glandular breast tissue proliferation seen in males, has most consistently been associated with the use of EFV, although d4T and ddI have also been implicated.^64,65^ This is not related to lipodystrophy. The onset occurs several months after initiation of ART and it may be bilateral or unilateral. The mechanism appears to be related to oestrogen receptor activation in breast tissues by EFV.^66^

It is important to exclude other common causes of gynaecomastia, such as other medications (including spironolactone, calcium channel blockers, metoclopramide). A serum testosterone is useful in excluding hypogonadism as a cause. If the serum testosterone is low, other appropriate investigations should be performed to identify the cause and manage accordingly. If the serum testosterone level is normal, then EFV should be substituted, bearing in mind the general principles of single drug substitutions (patients who are virologically suppressed should be switched to DTG or RPV).

Resolution of gynaecomastia is generally slow, taking months and may be incomplete in a small percentage.^67^ It is, therefore, important to manage the expectations of the patient in this regard.

## Immune reconstitution inflammatory syndrome

Approximately 10%–20% of patients who start ART with advanced immunosuppression experience clinical deterioration during the first months because of IRIS. Two forms are recognised:

- Unmasking IRIS occurs in patients who have an unrecognised OI when ART is initiated, and who then present with exaggerated inflammatory features of that infection during early ART because of it being ‘unmasked’ by recovering immunity.
- Paradoxical IRIS occurs in patients who are being treated for an OI when they start ART, but who develop an immune-mediated worsening or recurrence of features of that infection after starting ART.

Immune reconstitution inflammatory syndrome is most frequently described in association with TB and CM. Skin conditions such as molluscum contagiosum and Kaposi’s sarcoma may also worsen because of IRIS. The diagnosis of IRIS can be difficult, mainly because there is no confirmatory diagnostic test. Diagnosis relies on recognition of the characteristic clinical presentation, ensuring that OIs are correctly diagnosed, and excluding alternative causes for deterioration, such as drug resistance (e.g. MDR-TB). Case definitions for TB and cryptococcal IRIS have been published.^68,69^ It is important to ensure that the underlying OI is treated appropriately. ART should be continued, unless IRIS is life-threatening (e.g. neurological involvement in TB-IRIS with depressed level of consciousness). Corticosteroids have been shown to reduce morbidity and improve symptoms in paradoxical TB-IRIS,^70^ and can be used in mycobacterial and fungal forms of IRIS when other causes for deterioration have been excluded, and particularly when IRIS features are severe. For paradoxical TB-IRIS, prednisone can be commenced at a dose of 1.5 mg/kg/day and weaned over four weeks, but a longer course may be required if symptoms recur on weaning.^71^ Steroids should not be used in patients with Kaposi’s sarcoma.

## Support and counselling

The patient should be informed about the benefits of ART and that side effects are usually minor and transient, or manageable. The patient should be given a treatment plan, specifying the drugs to be used (with names and details including the appearance of each drug, when and how they are to be taken and a brief indication of anticipated side effects and toxicity).

Poor adherence results in the development of drug resistance. There is a bell-shaped relationship between adherence and resistance: patients with very poor resistance may not acquire resistance to certain drugs because of insufficient drug pressure to select for resistance. The patient should be encouraged to discuss drug-related issues with his or her clinician.

## Prophylaxis in patients receiving antiretroviral therapy

### Opportunistic infections

The use of appropriate prophylaxis (primary or secondary) is essential in patients initiating ART. In general, prophylaxis can be discontinued once the CD4+ count has increased to 200 cells/μL (but certain minimal durations of prophylaxis apply for secondary prophylaxis – local and international guidelines should be consulted).

### Isoniazid preventive therapy

Clinical trials conducted in SA and Cote d’Ivoire have shown that isoniazid preventive therapy (IPT) has an additive effect with ART in preventing incident TB in HIV-infected patients.^4,72^ In the SA trial, there was a 37% reduction in incident TB when patients receiving ART were prescribed IPT (vs placebo) for 12 months. This benefit applied irrespective of tuberculin skin test (TST) status, and the trial included patients established on ART. All patients receiving ART should be considered for IPT and screened for active TB by using a symptom screen^73^ – defer IPT and investigate for active TB if any of the four symptoms (current cough, fever, night sweats or weight loss) are present. Consider sputum TB culture in all patients with a CD4+ count < 200 cells/μL before IPT initiation where feasible. In patients receiving IPT, monitor for neuropathy and hepatitis symptoms. Routine ALT monitoring is not indicated, but request ALT if hepatitis symptoms occur. The duration of IPT depends on TST and ART status as outlined in Table 15.

**TABLE 15:** Indications for and duration of isoniazid preventive therapy.

TST	Pre-ART*	On ART
Not done	IPT for 6 months	IPT for 12 months
Negative	IPT not indicated	IPT for 12 months
Positive	IPT for at least 36 months	IPT for at least 36 months

Note: This would only apply in the case of a patient wishing to defer ART initiation.

IPT, isoniazid preventive therapy; TST, tuberculin skin test; ART, antiretroviral therapy.
